# National, regional, and state-level all-cause and cause-specific under-5 mortality in India in 2000–15: a systematic analysis with implications for the Sustainable Development Goals

**DOI:** 10.1016/S2214-109X(19)30080-4

**Published:** 2019-05-13

**Authors:** Li Liu, Yue Chu, Shefali Oza, Dan Hogan, Jamie Perin, Diego G Bassani, Usha Ram, Shaza A Fadel, Arvind Pandey, Neeraj Dhingra, Damodar Sahu, Pradeep Kumar, Richard Cibulskis, Brian Wahl, Anita Shet, Colin Mathers, Joy Lawn, Prabhat Jha, Rakesh Kumar, Robert E Black, Simon Cousens

**Affiliations:** a Department of Population Family and Reproductive Health, Johns Hopkins Bloomberg School of Public Health, Baltimore, MD, USA; b Institute for International Programs, Department of International Health, Johns Hopkins Bloomberg School of Public Health, Baltimore, MD, USA; c International Vaccine Access Center, Department of International Health, Johns Hopkins Bloomberg School of Public Health, Baltimore, MD, USA; d Department of Infectious Disease Epidemiology, London School of Hygiene & Tropical Medicine, London, UK; e Health Metrics and Measurement Cluster, World Health Organization, Geneva, Switzerland; f Global Malaria Programme, World Health Organization, Geneva, Switzerland; g Centre for Global Child Health, The Hospital for Sick Children, Department of Paediatrics, University of Toronto, Toronto, ON, Canada; h Dalla Lana School of Public Health, University of Toronto, Toronto, ON, Canada; i Department of Public Health and Mortality Studies, International Institute for Population Sciences, Mumbai, India; j National Institute of Medical Statistics (Indian Council of Medical Research), New Delhi, India; k National AIDS Control Organization, New Delhi, India; l United Nations Development Programme, New Delhi, India

## Abstract

**Background:**

India had the largest number of under-5 deaths of all countries in 2015, with substantial subnational disparities. We estimated national and subnational all-cause and cause-specific mortality among children younger than 5 years annually in 2000–15 in India to understand progress made and to consider implications for achieving the Sustainable Development Goal (SDG) child survival targets.

**Methods:**

We used a multicause model to estimate cause-specific mortality proportions in neonates and children aged 1–59 months at the state level, with causes of death grouped into pneumonia, diarrhoea, meningitis, injury, measles, congenital abnormalities, preterm birth complications, intrapartum-related events, and other causes. AIDS and malaria were estimated separately. The model was based on verbal autopsy studies representing more than 100 000 neonatal deaths globally and 16 962 deaths among children aged 1–59 months at the subnational level in India. By applying these proportions to all-cause deaths by state, we estimated cause-specific numbers of deaths and mortality rates at the state, regional, and national levels.

**Findings:**

In 2015, there were 25·121 million livebirths in India and 1·201 million under-5 deaths (under-5 mortality rate 47·81 per 1000 livebirths). 0·696 million (57·9%) of these deaths occurred in neonates. There were disparities in child mortality across states (from 9·7 deaths [Goa] to 73·1 deaths [Assam] per 1000 livebirths) and regions (from 29·7 deaths [the south] to 63·8 deaths [the northeast] per 1000 livebirths). Overall, the leading causes of under-5 deaths were preterm birth complications (0·330 million [95% uncertainty range 0·279–0·367]; 27·5% of under-5 deaths), pneumonia (0·191 million [0·168–0·219]; 15·9%), and intrapartum-related events (0·139 million [0·116–0·165]; 11·6%), with cause-of-death distributions varying across states and regions. In states with very high under-5 mortality, infectious-disease-related causes (pneumonia and diarrhoea) were among the three leading causes, whereas the three leading causes were all non-communicable in states with very low mortality. Most states had a slower decline in neonatal mortality than in mortality among children aged 1–59 months. Ten major states must accelerate progress to achieve the SDG under-5 mortality target, while 17 are not on track to meet the neonatal mortality target.

**Interpretation:**

Efforts to reduce vaccine-preventable deaths and to reduce geographical disparities should continue to maintain progress achieved in 2000–15. Enhanced policies and programmes are needed to accelerate mortality reduction in high-burden states and among neonates to achieve the SDG child survival targets in India by 2030.

**Funding:**

Bill & Melinda Gates Foundation.

## Introduction

An estimated 1·201 million deaths occurred in children younger than 5 years in India in 2015—the largest number of such deaths among all countries. The number reduced from 2·516 million in 2000,^1^ yet despite this progress, India did not meet target 4 of the Millennium Development Goals (MDG 4) of a two-thirds reduction in under-5 mortality rate between 1990 and 2015.^2^ During this period, there were substantial geographical disparities and socioeconomic inequalities in child survival within India.^3^, ^4^, ^5^

Information on national, regional, and state-level under-5 mortality rates and causes of deaths is of great value to guide health spending on child survival programmes. This is particularly true for India, with its decentralised health policy and decision making,^6^ increasing national health budget,^7^ and growing domestic and international interests in vaccine introduction and scaling up.^8^, ^9^, ^10^ We have previously published reports on district-level all-cause child mortality for 2001 and 2012,^11^ and on national and state-level causes of death in children for 2001–03^12^ and 2000–15,^5^ based on observed data from India's sample registration system. In this paper, as the Maternal and Child Epidemiology Estimation (MCEE) group, we provide estimates of all-cause and cause-specific under-5 mortality, with a focus on cause-specific mortality, at the national, regional, and state levels annually for 2000–15. We also reflect on progress made with regard to child survival during the MDG period, and draw implications for achieving the child survival targets (≤25 under-5 deaths per 1000 livebirths and ≤12 neonatal deaths per 1000 livebirths by 2030) in the Sustainable Development Goals (SDG) era in India.^13^

**Research in context**

**Evidence before this study**

We have been routinely reviewing and publishing studies on child mortality by cause estimates at global and national level since 2003. This study shared the same search strategies and screening criteria as our last publication for the 2000–15 period, with search terms covering child, mortality or death, and leading causes of deaths. We identified child cause-of-death studies published until Feb 12, 2015, in the following databases: PubMed, Embase, ISIS Web of Knowledge, Medline BIOSIS, Popline, WHOLIS, and IndMed. We have previously reported estimates of all-cause mortality at national and district levels for India for the years 2001 and 2012, as well as cause-specific child mortality estimates based on empirical data from the Million Deaths Study for major states and selected major causes for children younger than 5 years. The Global Burden of Disease Study has published mortality by cause among all age groups, including deaths among children younger than 5 years, in India. However, there are few data currently available for children younger than 5 years, especially at the subnational level, for the years 2000–15. Thus, this study aimed to fill this gap and provide more detailed modelled estimates by state, age, and cause.

**Added value of this study**

We present all-cause and cause-specific mortality estimates at state level annually for 2000–15 based on a comprehensive set of high-quality national and subnational verbal autopsy studies and a multicause model. We provide transparent time trends for neonates and children aged 1–59 months at national and subnational levels, reflect on achievements against Millennium Development Goal (MDG) 4, and highlight areas where greater efforts are required if states are to achieve the Sustainable Development Goal (SDG) 3 child survival targets by 2030.

**Implications of all the available evidence**

Information on child mortality by cause at the state, regional, and national levels shows that achievements with regard to child survival have been made in India in the MDG era, informing policy making and resource allocation in the SDG era. For example, efforts in integrated newborn care packages could further improve neonatal health outcomes. High and consistent commitment from local governments is crucial to reduce subnational disparities. Further work is required to improve the resolution of all-cause and cause-specific estimates to support decentralised decision making. Continued investment in high-quality and timely collection, dissemination, and use of child cause-of-death data at the subnational level in India is essential. Although some states are on track or have already achieved the SDG child mortality targets, ten of the 25 major states need to accelerate progress during 2015–30 to meet the under-5 mortality target, and 17 need to accelerate progress to meet the neonatal mortality target by 2030.

## Methods

### Estimation of state-level livebirths, under-5 all-cause mortality rates, and numbers of deaths in 2000–15

We largely followed our previously published methods to estimate livebirths, under-5 all-cause mortality rates, and number of deaths per state (including union territories).^11^ We extracted state-level population totals from 1991, 2001, and 2011 national censuses,^14^ and interpolated for years between two adjacent censuses and extrapolated for 2012–15, assuming exponential annual growth rates. We estimated state-level crude birth rates using data from the sample registration system.^15^, ^16^, ^17^ For crude birth rates in 2015, we assumed the same state-level trends as those in 2000–14 and imputed for states with missing values. We calculated the number of livebirths in each state by multiplying crude birth rates by total population, and rescaled to match the national total number of livebirths estimated by the UN Population Division.^18^

We derived state-level neonatal mortality rates using similar methods and data sources to those used for crude birth rate, with missing values imputed on the basis of the relative relationship of infant mortality rate among states. State-level under-5 mortality rates were obtained with one of two approaches. For 20 large states, we used the under-5 mortality rate from the sample registration system,^16^ and calculated the rates for missing years before 2008 using infant mortality rate and age-specific death rates at 1–4 years of age. For the remaining 15 states and union territories, we selected states with available data as references and, for those without data, imputed under-5 mortality rates on the basis of the ratio of infant mortality to under-5 mortality in the reference state for each year. We calculated the number of neonatal and under-5 deaths in each state using state-level neonatal mortality rate or under-5 mortality rate and livebirth data, and rescaled to the national totals estimated by the UN Inter-Agency Group for Child Mortality Estimation.^19^ Additional details are available in the appendix (pp 4–6).

### Estimation of national, regional, and state-level cause-specific mortality proportions in 2000–15

State-level cause-specific mortality proportions for neonates and children aged 1–59 months were estimated separately with multinomial logistic regression models using data from verbal autopsy studies. The analytical framework has been published elsewhere.^1^, ^20^, ^21^, ^22^, ^23^, ^24^

The model input data were different for the two age groups. For neonates, input data from all countries with high mortality where verbal autopsy studies were available were used.^1^ The rationale for using verbal autopsy studies from all countries with high mortality is provided in the appendix (p 7). 124 verbal autopsy datapoints were included, covering more than 100 000 neonatal deaths across 37 countries. After the model had been parameterised, state covariates were used to predict state-level neonatal cause-specific mortality proportions.

For children who died at between 1 month and 59 months of age, we developed a subnational verbal-autopsy-based multicause model, using only subnational verbal autopsy studies from India to best account for the cause-of-death profile in this unique setting (appendix pp 7–8). Verbal autopsy studies were identified through a comprehensive literature review.^1^, ^24^ Unpublished high-quality verbal autopsy studies were identified by contacting known investigators. 50 datapoints from 16 subnational verbal autopsy studies across 23 states were used, representing 16 962 deaths. We also extracted study covariate values when available in the verbal autopsy studies, or else used estimates of the highest possible geographical time resolution from other sources.^4^, ^25^, ^26^

We excluded deaths due to AIDS and malaria in the multinomial model because they were difficult to estimate stably because of the low disease burden. Instead, AIDS and malaria were estimated separately. AIDS deaths per state were estimated by the Indian Council of Medical Research using Spectrum, an analytical software that has been routinely used by UNAIDS. Malaria deaths per state were estimated by the WHO Malaria Programme on the basis of reported malaria cases and case fatality rates.^27^ Details of AIDS and malaria death estimation are provided in the appendix (pp 8–9). We applied the state share of deaths due to AIDS and malaria to national estimates produced by UNAIDS and WHO, respectively, to derive state-level estimates.

We grouped the remaining causes of death into the following categories, based on International Classification of Diseases 10th revision:^24^ pneumonia, diarrhoea, meningitis, injury, measles, congenital abnormalities, causes originating in the perinatal period (ie, preterm birth complications and intrapartum-related events), and other. Ordinary least-squares regression was used to identify possible covariates for the log-ratio of the proportion of each of the seven causes relative to pneumonia (reference). The final model was selected on the basis of results from cross-validation.^1^ We then fitted the multinomial logistic regression model with robust SEs to obtain parameter estimates, with study datapoints weighted proportionally to the inverse of the square root of the total number of deaths.

After the verbal-autopsy-based multicause model had been parameterised, we used state covariates to predict state-level cause-specific mortality proportions. Details of how the state prediction covariate database was prepared and the final covariates retained are provided in the appendix (p 11). We then applied the age-specific and state-specific cause-specific mortality proportions to numbers of deaths (excluding those due to AIDS and malaria) to derive age-specific and cause-specific numbers of deaths. Among children who died between the ages of 1 month and 59 months, to account for the effects of the recent scaling up (ie, up to 2015) of the *Haemophilus influenzae* type b (Hib) vaccine on deaths due to pneumonia and meningitis, we included a post-hoc adjustment, details of which have been published previously^1^, ^23^ (for a summary see appendix pp 11–12).

We applied the state-level cause-specific mortality proportions by age to state-specific and age-specific all-cause deaths to derive age-specific and cause-specific numbers of deaths annually from 2000 to 2015 for each of the 35 states. An overview of the estimation process is shown in the appendix (p 13). Uncertainty ranges were calculated with use of bootstrap resampling of the input data with replacement.^1^ The 2·5th and 97·5th percentiles were taken as the lower and upper limits of the uncertainty.

### Estimates reporting, comparison, and transparency

We report deaths by cause for each of the 23 major states, with the 12 smaller states grouped into northeastern states (Arunachal Pradesh, Manipur, Mizoram, Nagaland, Sikkim, and Tripura) and other states (Andaman and Nicobar Islands, Chandigarh, Dadra and Nagar Haveli, Daman and Diu, Lakshadweep, and Puducherry) on the basis of geographical location (appendix p 14). Small states were defined as those with fewer than 1500 deaths in 2015, except for Goa. We report Andhra Pradesh and Telangana together in 2014 and 2015 because Telangana was separated from Andhra Pradesh in June, 2014. The states of Bihar, Chhattisgarh, Madhya Pradesh, Odisha, Rajasthan, Uttar Pradesh, Jharkhand, and Uttaranchal, which are the least developed and lag behind the rest of India in terms of demographic transition, constitute the Empowered Action Group (EAG).^28^ Together with Assam, they are referred to as the EAGA states.

We report estimates nationally and by state, region (north, northeast, east, central, west, and south), and EAGA status (appendix p 14). We also classified states into five strata of under-5 mortality rate: very high mortality (>65 deaths per 1000 livebirths), high mortality (>55 to 65 deaths per 1000 livebirths), medium mortality (>45 to 55 deaths per 1000 livebirths), low mortality (>25 to 45 deaths per 1000 livebirths), and very low mortality (≤25 deaths per 1000 livebirths; see appendix p 14 for groupings). Annual rates of reduction^24^ were used to describe the pace of decline in mortality, and were benchmarked against 4·4%—the annual rate of reduction required to reach MDG 4 (appendix p 12).

We compared our estimates with other estimates for the same period based on the 2000–15 Million Death Study (MDS)^5^ and the 1980–2016 Global Burden of Disease (GBD) study.^29^ Constrained by estimate availability and accessibility, we were only able to compare cause-specific mortality proportions for 2015 at the national level across the three sources, and compared trends in cause-specific mortality rates between MCEE and MDS at the national and state levels.

Our study conformed to the Guidelines for Accurate and Transparent Health Estimates Reporting (GATHER) statement (appendix p 15).^30^ Analyses were done with Stata software (version 15.1).

### Role of the funding source

The funder of the study had no role in study design, data collection, data analysis, data interpretation, or writing of the report. All authors had full access to all the data in the study, and the corresponding author had final responsibility for the decision to submit for publication.

## Results

In 2015, an estimated 25·121 million children were born in India, and 1·201 million children died before the age of 5 years, equating to an under-5 mortality rate of around 47·81 per 1000 livebirths (table 1). 0·696 million (57·9%) of these under-5 deaths occurred in the neonatal period (neonatal mortality rate 27·70 per 1000 livebirths). Large disparities existed across regions, with the highest under-5 mortality rates in the northeast region (63·8 deaths per 1000 livebirths) and central region (60·6 deaths per 1000 livebirths), both more than twice that of the south region (29·7 deaths per 1000 livebirths). After the south region, the lowest rates were observed in the west (31·8 deaths per 1000 livebirths) and north (35·2 deaths per 1000 livebirths) regions, increasing to 49·3 deaths per 1000 livebirths in the east region. Under-5 mortality rate in EAGA states (58·7 deaths per 1000 livebirths) was more than 1·8 times that of the non-EAGA states (32·1 deaths per 1000 livebirths).

**Table 1:** Estimated numbers of livebirths and infant and child deaths at the national, regional, and state levels in India in 2015

		**Total livebirths**	**All under-5 deaths**		**Deaths in infants aged 0–28 days**		**Deaths in children aged 1–59 months**	
			Total	Per 1000 livebirths	Total	Per 1000 livebirths	Total	Per 1000 livebirths
India		25 121 029	1 200 998	47·81	695 852	27·70	505 146	20·11
Region								
	Central	9 512 025	575 973	60·55	328 335	34·52	247 638	26·03
	East	5 823 480	286 833	49·25	166 061	28·52	120 773	20·74
	North	1 733 503	60 924	35·15	36 932	21·30	23 992	13·84
	Northeast	932 280	59 446	63·76	22 547	24·18	36 899	39·58
	South	4 046 896	120 126	29·68	79 772	19·71	40 354	9·97
	West	3 072 844	97 696	31·79	62 206	20·24	35 490	11·55
State								
	Andhra Pradesh and Telangana	1 409 102	55 920	39·68	35 392	25·12	20 528	14·57
	Assam	685 611	50 129	73·12	18 694	27·27	31 435	45·85
	Bihar	2 832 353	148 484	52·42	83 026	29·31	65 458	23·11
	Chhattisgarh	620 865	32 267	51·97	19 339	31·15	12 928	20·82
	Delhi	280 798	6869	24·46	4583	16·32	2286	8·14
	Goa	17 485	170	9·72	115	6·58	55	3·15
	Gujarat	1 256 413	54 095	43·06	32 738	26·06	21 357	17·00
	Haryana	526 637	22 645	43·00	13 482	25·60	9163	17·40
	Himachal Pradesh	103 513	4133	39·93	2536	24·50	1598	15·44
	Jammu and Kashmir	217 654	8503	39·07	6412	29·46	2091	9·61
	Jharkhand	809 086	37 592	46·46	22 431	27·72	15 160	18·74
	Karnataka	1 047 947	34 038	32·48	24 218	23·11	9820	9·37
	Kerala	466 438	5832	12·50	2930	6·28	2902	6·22
	Madhya Pradesh	1 859 145	124 693	67·07	66 916	35·99	57 776	31·08
	Maharashtra	1 784 820	42 954	24·07	29 063	16·28	13 891	7·78
	Meghalaya	72 388	4465	61·68	1665	23·00	2800	38·68
	Northeastern states*	174 281	4852	27·84	2188	12·55	2664	15·29
	Odisha	785 330	50 363	64·13	30 000	38·20	20 363	25·93
	Other states†	53 096	1275	24·01	799	15·05	476	8·96
	Punjab	412 754	11 483	27·82	5712	13·84	5771	13·98
	Rajasthan	1 708 901	93 513	54·72	54 430	31·85	39 082	22·87
	Tamil Nadu	1 098 325	23 849	21·71	16 905	15·39	6944	6·32
	Uttar Pradesh	5 323 115	325 500	61·15	187 649	35·25	137 851	25·90
	Uttarakhand	178 264	6980	39·16	4024	22·57	2956	16·58
	West Bengal	1 396 712	50 395	36·08	30 604	21·91	19 792	14·17

* Arunachal Pradesh, Manipur, Mizoram, Nagaland, Sikkim, and Tripura.

† Andaman and Nicobar Islands, Chandigarh, Dadra and Nagar Haveli, Daman and Diu, Lakshadweep, and Puducherry.

**Figure 1:**
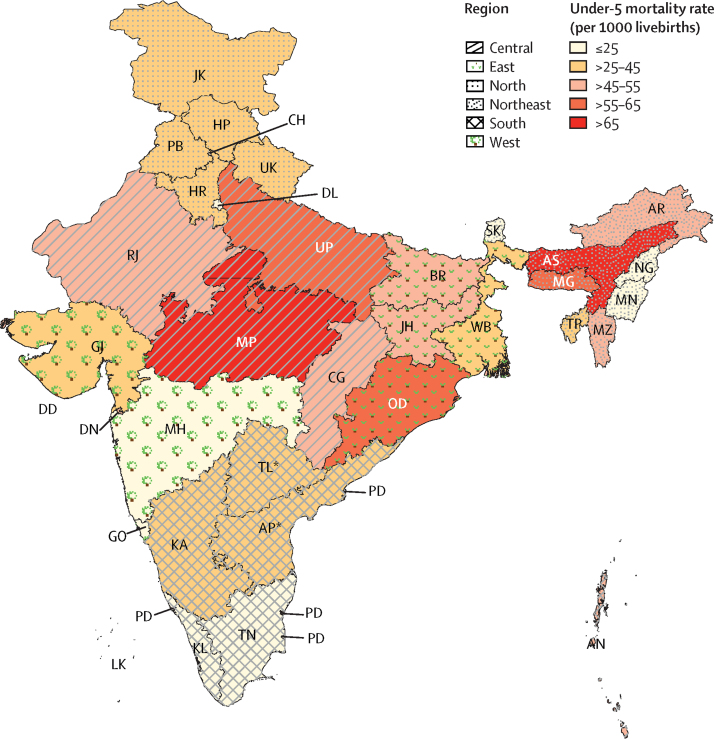
Under-5 mortality by state in 2015 AN=Andaman and Nicobar. AP=Andhra Pradesh. AR=Arunachal Pradesh. AS=Assam. BR=Bihar. CG=Chhattisgarh. CH=Chandigarh. DD=Daman and Diu. DL=National Capital Territory of Delhi. DN=Dadra and Nagar Haveli. GO=Goa. GJ=Gujarat. HR=Haryana. HP=Himachal Pradesh. JK=Jammu and Kashmir. JH=Jharkhand. KA=Karnataka. KL=Kerala. LK=Lakshadweep. MG=Meghalaya. MH=Maharashtra. MN=Manipur. MP=Madhya Pradesh. MZ=Mizoram. NG=Nagaland. OD=Odisha. PB=Punjab. PD=Puducherry. RJ=Rajasthan. SK=Sikkim. TL=Telangana. TN=Tamil Nadu. TP=Tripura. UK=Uttarakhand. UP=Uttar Pradesh. WB=West Bengal. *TL is reported together with AP.

States with the highest burden (numbers) of under-5 deaths were mostly those clustered in the central and east regions, with half of all under-5 deaths occurring in three states: Uttar Pradesh, Bihar, and Madhya Pradesh (table 1). A similar clustering pattern was observed for neonatal death burden. The highest under-5 mortality rates were in Assam (73·1 per 1000 livebirths), Madhya Pradesh (67·1 per 1000 livebirths), and Odisha (64·1 per 1000 livebirths), whereas the lowest rates were in Goa (9·7 per 1000 livebirths), Kerala (12·5 per 1000 livebirths) and Tamil Nadu (21·7 per 1000 livebirths; figure 1). The proportion of neonatal deaths (out of all under-5 deaths) ranged from 37·3% (in Meghalaya) to 75·4% (in Jammu and Kashmir).

**Figure 2:**
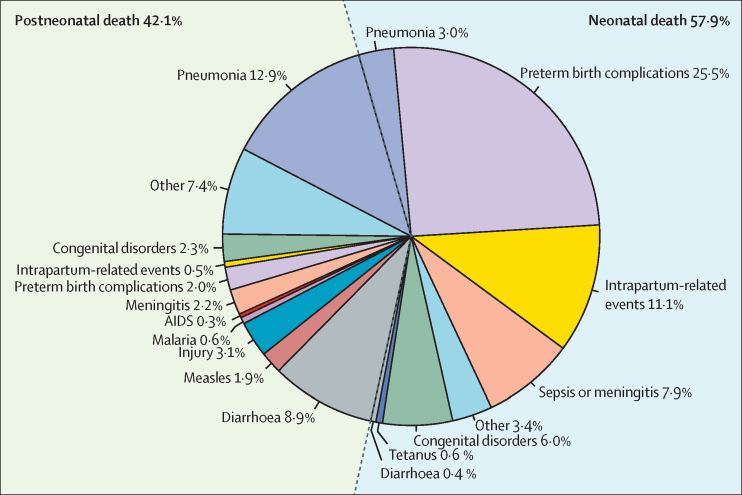
Distribution of causes of under-5 deaths in 2015 Causes are separated by age group (<1 month and 1–59 months).

Nationally, the leading causes of under-5 deaths in 2015 were preterm birth complications (0·330 million [95% uncertainty range 0·279–0·367]; 27·5% of under-5 deaths), pneumonia (0·191 million [0·168–0·219]; 15·9%), and intrapartum-related events (0·139 million [0·116–0·165]; 11·6%). Among neonates, the leading causes were preterm birth complications (0·306 million [0·257–0·343]; 44·0% of all neonatal deaths), intrapartum-related events (0·133 million [0·110–0·158]; 19·1%), and neonatal sepsis or meningitis (0·095 million [0·062–0·130]; 13·7%; table 2, figure 2). Among children who died between 1 month and 59 months of age, pneumonia (0·155 million [0·138–0·172]; 30·6%), diarrhoea (0·107 million [0·096–0·117]; 21·3%), and injuries (0·037 million [0·030–0·043]; 7·3%) were the leading causes (table 3, figure 2).

**Table 2:** Estimated numbers of cause-specific deaths in infants aged 0–28 days at the national, regional, and state levels in India in 2015

		**Preterm birth complications**	**Intrapartum-related events**	**Sepsis or meningitis**	**Congenital**	**Pneumonia**	**Tetanus**	**Injury**	**Diarrhoea**	**Other disorders**
India		306 462 (256 814–342 719),25·5%	132 900 (109 692–158 231), 11·1%	95 046 (62 027–130 094), 7·9%	71 802 (56 681–92 640), 6·0%	36 151 (21 709–58 758), 3·0%	6788 (3314–16 655), 0·6%	5472 (2814–8014), 0·5%	5331 (2898–28 312),0·4%	35 900 (18 480–52 560), 3·0%
Region										
	Central	157 218 (128 320–177 767),27·3%	58 855 (46 924–71 605),10·2%	43 783 (27 020–62 189), 7·6%	25 671 (19 970–36 038), 4·5%	16 906 (10 383–27 229), 2·9%	4158 (2036–9964), 0·7%	2475 (1148–3809), 0·4%	3037 (1605–18 758), 0·5%	16 232 (7540–24 971),2·8%
	East	70 525 (58 380–80 167),24·6%	32 730 (26 737–39 318), 11·4%	24 695 (15 993–33 819), 8·6%	16 284 (12 742–21 227), 5·7%	8679 (5297–13 943), 3·0%	1453 (684–3753), 0·5%	1374 (688–2094), 0·5%	1303 (696–6422), 0·5%	9018 (4520–13 733),3·1%
	North	14 077 (12 268–15 611),23·1%	7867 (6730–9115), 12·9%	4837 (3306–6421), 7·9%	5480 (4073–6997), 9·0%	1989 (1129–3370),3·3%	240 (113–627), 0·4%	299 (180–399), 0·5%	184 (97–778), 0·3%	1959 (1182–2614),3·2%
	Northeast	10 171 (8739–11 342),17·1%	4628 (3877–5360), 7·8%	2891 (1845–4011), 4·9%	1828 (1484–2529),3·1%	1245 (765–2002),2·1%	183 (86–478), 0·3%	186 (99–274), 0·3%	193 (104–788), 0·3%	1222 (650–1797),2·1%
	South	28 114 (22 523–33 522),23·4%	17 047 (13 729–20 786), 14·2%	11 346 (7678–15 770), 9·4%	13 612 (9470–17 631), 11·3%	4042 (2045–7472), 3·4%	325 (136–910), 0·3%	667 (390–916), 0·6%	242 (80–1294), 0·2%	4377 (2569–6014),3·6%
	West	26 357 (22 713–29 698),27·0%	11 774 (9959–14 224),12·1%	7494 (4935–10 227), 7·7%	8927 (6206–11 589), 9·1%	3290 (1984–5641), 3·4%	430 (206–1167), 0·4%	471 (248–687), 0·5%	372 (233–960), 0·4%	3091 (1633–4507),3·2%
State										
	Andhra Pradesh and Telangana	12 390 (9472–15 241),22·2%	7822 (6105–9591),14·0%	5520 (3706–7670), 9·9%	5283 (3942–6871), 9·4%	1789 (895–3288), 3·2%	157 (64–436), 0·3%	306 (175–426), 0·5%	119 (27–836), 0·2%	2007 (1148–2796), 3·6%
	Assam	8740 (7353–9847), 17·4%	3668 (3014–4355), 7·3%	2470 (1541–3448), 4·9%	1290 (1004–1953), 2·6%	1024 (633–1648), 2·0%	149 (67–402), 0·3%	156 (71–242), 0·3%	176 (94–723), 0·4%	1021 (466–1588),2·0%
	Bihar	36 289 (28 632–42 417),24·4%	16 280 (12 797–20 048), 11·0%	12 291 (7591–16 952),8·3%	7381 (5637–10 029), 5·0%	4321 (2692–6859), 2·9%	786 (349–2157), 0·5%	656 (321–1034), 0·4%	718 (377–3210), 0·5%	4304 (2111–6786), 2·9%
	Chhattisgarh	6951 (5670–7915), 21·5%	4527 (3756–5325),14·0%	3164 (2126–4125), 9·8%	1980 (1561–2636), 6·1%	1071 (666–1700), 3·3%	204 (97–525), 0·6%	172 (98–244), 0·5%	143 (59–1015), 0·4%	1128 (644–1602), 3·5%
	Delhi	1832 (1523–2207),26·7%	817 (670–999), 11·9%	667 (446–989), 9·7%	672 (396–913),9·8%	240 (146–414), 3·5%	<30, 0·2%	42 (17–62), 0·6%	<30, 0·4%	274 (111–405), 4·0%
	Goa	40 (30–50), 23·5%	<30, 13·5%	<30, 6·5%	<30, 17·0%	<30, 3·5%	<30,0·0%	<30, 0·6%	<30, 0·0%	<30, 2·9%
	Gujarat	15 203 (12 330–17 241), 28·1%	5976 (4866–7418),11·0%	4094 (2492–5871), 7·6%	3189 (2386–4327),5·9%	1787 (1181–2970), 3·3%	332 (160–912), 0·6%	248 (100–410), 0·5%	282 (168–762), 0·5%	1627 (657–2692),3·0%
	Haryana	5250 (4494–5979), 23·2%	2944 (2443–3398),13·0%	1646 (1087–2206), 7·3%	1966 (1522–2545), 8·7%	731 (383–1280), 3·2%	101 (45–272), 0·4%	103 (63–137), 0·5%	62 (24–333), 0·3%	677 (413–895), 3·0%
	Himachal Pradesh	1031 (903–1151), 24·9%	523 (452–605),12·7%	283 (182–397), 6·8%	382 (291–492), 9·2%	137 (72–250), 3·3%	<30 (7–38), 0·4%	<30 (12–28), 0·5%	<30 (6–48), 0·3%	133 (77–182), 3·2%
	Jammu and Kashmir	2094 (1685–2414), 24·6%	1554 (1293–1848),18·3%	1015 (708–1304), 11·9%	876 (659–1133), 10·3%	353 (212–588), 4·2%	66 (31–168), 0·8%	55 (33–78), 0·6%	35 (12–266), 0·4%	363 (216–514), 4·3%
	Jharkhand	10 246 (8097–11 630), 27·3%	4160 (3321–5167), 11·1%	3001 (1767–4503), 8·0%	2013 (1512–2767),5·4%	1215 (797–1996), 3·2%	206 (95–566), 0·5%	183 (79–304), 0·5%	203 (116–647), 0·5%	1202 (522–1996),3·2%
	Karnataka	8790 (7049–10 329), 25·8%	5160 (4178–6347), 15·2%	3291 (2220–4496), 9·7%	4086 (2899–5306),12·0%	1242 (622–2298), 3·6%	117 (51–316),0·3%	193 (113–276), 0·6%	73 (25–358), 0·2%	1265 (742–1811),3·7%
	Kerala	936 (757–1179), 16·0%	587 (472–745), 10·1%	354 (228–540), 6·1%	703 (383–945), 12·1%	145 (71–293), 2·5%	<30, 0·1%	<30, 0·4%	<30,0·1%	170 (94–233), 2·9%
	Madhya Pradesh	35 503 (27 928–40 886),28·5%	10 400 (7762–13 161), 8·3%	7694 (4358–11 463),6·2%	5317 (3868–8210),4·3%	3278 (1769–5748),2·6%	762 (363–1990),0·6%	4470 (187–746), 0·4%	582 (291–3671), 0·5%	2932 (1226–4894), 2·4%
	Maharashtra	11 007 (9411–12 685),25·6%	5710 (4778–6936), 13·3%	3349 (2225–4646), 7·8%	5671 (3570–7443), 13·2%	1480 (758–2835), 3·4%	95 (39–282), 0·2%	220 (131–304), 0·5%	88 (46–192), 0·2%	1443 (862–1991), 3·4%
	Meghalaya	654 (550–750), 14·6%	430 (358–486), 9·6%	179 (113–249), 4·0%	173 (141–233), 3·9%	98 (57–156), 2·2%	<30, 0·4%	<30, 0·3%	<30, 0·2%	89 (56–116), 2·0%
	Northeastern states*	777 (660–892), 16·0%	529 (442–627), 10·9%	242 (158–330), 5·0%	365 (255–475),7·5%	123 (72–202), 2·5%	<30, 0·3%	<30,0·4%	<30, 0·2%	112 (74–150), 2·3%
	Odisha	13 368 (10 920–15 194),26·5%	5653 (4561–6663),11·2%	4444 (2828–6134), 8·8%	2460 (1913–3513),4·9%	1564 (911–2549), 3·1%	346 (171–810),0·7%	250 (110–388), 0·5%	271 (119–2132), 0·5%	1644 (722–2547), 3·3%
	Other states†	305 (261–349), 23·9%	162 (137–197),12·7%	93 (61–124), 7·3%	145 (93–191), 11·4%	42 (25–75), 3·3%	<30, 0·3%	<30, 0·5%	<30, 0·3%	39 (23–55), 3·1%
	Punjab	2118 (1794–2451), 18·4%	1188 (990–1430), 10·3%	666 (446–905), 5·8%	1083 (726–1419), 9·4%	299 (152–555), 2·6%	<30, 0·2%	43 (26–58), 0·4%	<30, 0·1%	282 (172–383), 2·5%
	Rajasthan	30 402 (22 711–34 993), 32·5%	7244 (5129–10 185),7·7%	6784 (3782–10 781),7·3%	4089 (2917–6081), 4·4%	2490 (1359–4529), 2·7%	490 (236–1238), 0·5%	342 (126–641), 0·4%	347 (146–1712), 0·4%	2243 (831–4207), 2·4%
	Tamil Nadu	5873 (5003–7020),24·6%	3414 (2829–4113),14·3%	2148 (1446–3096), 9·0%	3470 (2062–4592), 14·6%	850 (441–1572), 3·6%	45 (17–139), 0·2%	140 (83–185), 0·6%	44 (22–96), 0·2%	921 (547–1212), 3·9%
	Uttar Pradesh	84 362 (68 772–95 759),25·9%	36 685 (29 468–43 882),11·3%	26 140 (16 241–36 674), 8·0%	14 286 (11 010–20 026), 4·4%	10 066 (6461–15 811), 3·1%	2702 (1329–6427), 0·8%	1514 (722–2220), 0·5%	1965 (1025–12 211), 0·6%	9928 (4736–14 553), 3·1%
	Uttarakhand	1677 (1429–1919),24·0%	805 (679–948),11·5%	541 (354–749), 7·8%	465 (343–606), 6·7%	221 (142–355), 3·2%	<30, 0·4%	34 (16–49), 0·5%	33 (20–77), 0·5%	222 (108–320), 3·2%
	West Bengal	10 622 (8927–12 207),21·1%	6637 (5574–7875),13·2%	4959 (3375–6854), 9·8%	4430 (3219–5670), 8·8%	1578 (868–2723), 3·1%	114 (46–341), 0·2%	285 (155–395), 0·6%	111 (46–424), 0·2%	1869 (1018–2590), 3·7%

Data are n (95% uncertainty interval), % of total under-5 deaths.

* Arunachal Pradesh, Manipur, Mizoram, Nagaland, Sikkim, and Tripura.

† Andaman and Nicobar Islands, Chandigarh, Dadra and Nagar Haveli, Daman and Diu, Lakshadweep, and Puducherry.

**Table 3:** Estimated numbers of cause-specific deaths in children aged 1–59 months at the national, regional, and state levels in India in 2015

		**Pneumonia**	**Diarrhoea**	**Injury**	**Congenital**	**Meningitis**	**Preterm birth complications**	**Measles**	**Malaria**	**Intrapartum-related events**	**HIV**	**Other disorders**
India		154 511 (138 493–172 048), 12·9%	107 415 (96 216–117 286), 8·9%	36 924 (30 387–43 340), 3·1%	28 035 (21 820–33 777), 2·3%	26 001 (21 085–32 019), 2·2%	23 536 (15 817–30 438), 2·0%	22 703 (14 319–34 250), 1·9%	6627 (2429–12 557), 0·6%	5884 (3954–7610), 0·5%	4036 (3213–4951), 0·3%	89 474 (80 529–98 796), 7·4%
Region												
	Central	77 936 (70 535–85 551), 13·5%	58 164 (53 442–62 807), 10·1%	15 228 (12 930–17 353), 2·6%	9764 (7744–11 660), 1·7%	13 119 (10 598–16 274), 2·3%	11 049 (7710–13 974), 1·9%	11 908 (7499–18 010), 2·1%	1345 (493–2548), 0·2%	2762 (1927–3494), 0·5%	1249 (986–1448), 0·2%	45 112 (40 637–49 902), 7·8%
	East	36 651 (32 603–40 785), 12·8%	26 046 (23 324–28 366), 9·1%	7945 (6629–9197), 2·8%	5412 (4286–6423), 1·9%	6165 (4956–7584), 2·1%	5752 (3750–7508), 2·0%	5033 (3087–7727), 1·8%	3856 (1413–7306), 1·3%	1438 (937–1877), 0·5%	1238 (1075–1660), 0·4%	21 236 (19 005–23 497), 7·4%
	North	6874 (5998–7961), 11·3%	3756 (2851–4542), 6·2%	2583 (1801–3383), 4·2%	2472 (1643–3182),4·1%	1156 (940–1429), 1·9%	1245 (741–1698), 2·0%	1057 (659–1632), 1·7%	358 (131–679), 0·6%	311 (185–425), 0·5%	198 (126–263), 0·3%	3983 (3549–4490), 6·5%
	Northeast	11 530 (10 300–12 891), 19·4%	7221 (6091–8132), 12·1%	3178 (2507–3820), 5·3%	2465 (1892–2982), 4·1%	1940 (1579–2391), 3·3%	1507 (1082–1874), 2·5%	1646 (1039–2486), 2·8%	233 (85–441), 0·4%	377 (270–469), 0·6%	125 (86–184), 0·2%	6678 (6070–7391),11·2%
	South	11 107 (9453–13 284), 9·2%	5563 (3901–7058), 4·6%	5019 (3335–6863), 4·2%	5403 (3278–7260), 4·5%	1868 (1503–2302), 1·6%	2088 (1205–2897), 1·7%	1545 (952–2356), 1·3%	193 (71–365), 0·2%	522 (301–724), 0·4%	612 (481–704), 0·5%	6435 (5604–7383), 5·4%
	West	10 413 (9212–11 774), 10·7%	6664 (5675–7501), 6·8%	2972 (2303–3627), 3·0%	2519 (1849–3115), 2·6%	1752 (1419–2152), 1·8%	1895 (1114–2593), 1·9%	1514 (955–2276), 1·5%	642 (235–1217), 0·7%	474 (278–648), 0·5%	614 (459–693), 0·6%	6029 (5412–6698), 6·2%
State												
	Andhra Pradesh and Telangana	5602 (4738–6741), 10·0%	2743 (1881–3526), 4·9%	2651 (1734–3677), 4·7%	2937 (1746–3986), 5·3%	942 (755–1168), 1·7%	980 (597–1330), 1·8%	786 (484–1194), 1·4%	73 (27–139), 0·1%	245 (149–332), 0·4%	324 (273–381), 0·6%	3245 (2801–3748), 5·8%
	Assam	9903 (8850–11 069), 19·8%	6180 (5208–6966), 12·3%	2750 (2166–3313), 5·5%	2141 (1646–2594), 4·3%	1666 (1353–2054), 3·3%	1245 (900–1539), 2·5%	1365 (841–2087), 2·7%	110 (40–208), 0·2%	311 (225–385), 0·6%	<30, 0·1%	5737 (5218–6339), 11·4%
	Bihar	20 731 (18 756–22 734), 14·0%	15 966 (14 773–17 180), 10·8%	3628 (2974–4160), 2·4%	2083 (1528–2539), 1·4%	3487 (2817–4327), 2·3%	3204 (2129–4131), 2·2%	2891 (1771–4409), 1·9%	66 (24–126), 0·0%	801 (532–1033), 0·5%	587 (588–879), 0·4%	12 015 (10 835–13 297), 8·1%
	Chhattisgarh	3930 (3471–4379), 12·2%	2935 (2634–3195), 9·1%	735 (605–845), 2·3%	441 (330–535), 1·4%	661 (529–819), 2·0%	609 (399–791), 1·9%	516 (306–824), 1·6%	604 (221–1144),1·9%	152 (100–198), 0·5%	69 (62–108), 0·2%	2277 (2022–2526), 7·1%
	Delhi	675 (597–770), 9·8%	397 (317–463), 5·8%	214 (161–266), 3·1%	182 (132–225), 2·6%	114 (93–139), 1·7%	137 (74–197), 2·0%	96 (61–144), 1·4%	<30, 0·0%	34 (18–49), 0·5%	44 (26–71),0·6%	391 (353–437), 5·7%
	Goa	<30,5·9%	<30, 2·9%	<30, 3·5%	<30, 4·1%	<30, 1·2%	<30, 1·2%	<30, 0·6%	<30, 7·6%	<30, 0·6%	<30, 1·8%	<30, 3·5%
	Gujarat	6519 (5832–7240),12·1%	4646 (4173–5042), 8·6%	1351 (1140–1545), 2·5%	867 (677–1034), 1·6%	1097 (885–1356), 2·0%	1103 (686–1478), 2·0%	969 (612–1457),1·8%	350 (128–663), 0·6%	276 (171–369), 0·5%	403 (316–454), 0·7%	3776 (3390–4180), 7·0%
	Haryana	2567 (2219–2977), 11·3%	1383 (1030–1693),6·1%	985 (677–1296), 4·3%	950 (622–1227), 4·2%	432 (345–534), 1·9%	435 (272–587), 1·9%	425 (252–704), 1·9%	333 (122–631), 1·5%	109 (68–147), 0·5%	56 (37–69), 0·2%	1488 (1312–1681), 6·6%
	Himachal Pradesh	474 (415–546), 11·5%	263 (203–315), 6·4%	170 (122–219), 4·1%	156 (107–199), 3·8%	80 (65–99), 1·9%	83 (50–112), 2·0%	66 (41–100), 1·6%	<30, 0·0%	<30, 0·5%	<30, 0·2%	274 (247–308), 6·6%
	Jammu and Kashmir	597 (517–702), 7·0%	308 (223–385), 3·6%	252 (170–338), 3·0%	258 (162–342), 3·0%	100 (81–124), 1·2%	105 (64–142), 1·2%	92 (57–141), 1·1%	<30,0·0%	<30, 0·3%	<30, 0·1%	346 (306–393), 4·1%
	Jharkhand	3816 (2827–4682), 10·2%	2640 (1932–3183), 7·0%	845 (602–1043), 2·2%	566 (387–712), 1·5%	642 (448–839), 1·7%	626 (359–862), 1·7%	530 (327–807), 1·4%	2971 (1089–5628),7·9%	156 (90–215), 0·4%	158 (118–189), 0·4%	2211 (1636–2673), 5·9%
	Karnataka	2764 (2377–3266), 8·1%	1438 (1047–1784), 4·2%	1146 (784–1532), 3·4%	1162 (740–1532), 3·4%	465 (377–572), 1·4%	520 (301–722), 1·5%	362 (219–572), 1·1%	82 (30–155), 0·2%	130 (75–180), 0·4%	149 (108–158), 0·4%	1602 (1413–1818), 4·7%
	Kerala	771 (643–944), 13·2%	372 (249–487), 6·4%	378 (246–525), 6·5%	428 (253–581), 7·3%	130 (103–160), 2·2%	176 (86–265), 3·0%	100 (61–160), 1·7%	<30, 0·1%	44 (22–66), 0·8%	52 (39–67), 0·9%	447 (381–522), 7·7%
	Madhya Pradesh	18 415 (16 541–20 346), 14·8%	12 575 (11 187–13 760), 10·1%	4197 (3501–4844), 3·4%	2869 (2265–3410), 2·3%	3097 (2509–3818), 2·5%	2461 (1761–3073), 2·0%	2517 (1538–3878), 2·0%	230 (84–435), 0·2%	615 (440–768), 0·5%	131 (105–152), 0·1%	10 671 (9675–11 776), 8·6%
	Maharashtra	3831 (3286–4539), 8·9%	1984 (1433–2476), 4·6%	1597 (1083–2140), 3·7%	1628 (1034–2138), 3·8%	645 (521–795), 1·5%	780 (422–1115), 1·8%	537 (333–813), 1·3%	271 (99–513), 0·6%	195 (105–279), 0·5%	207 (140–226), 0·5%	2217 (1942–2534), 5·2%
	Meghalaya	866 (773–964),19·4%	565 (486–630), 12·7%	217 (176–255), 4·9%	158 (123–189), 3·5%	146 (117–181), 3·3%	122 (85–155), 2·7%	152 (87–267), 3·4%	43 (16–82), 1·0%	<30,0·7%	<30, 0·0%	501 (449–556), 11·2%
	Northeastern states*	761 (668–861), 15·7%	475 (395–543), 9·8%	212 (165–255), 4·4%	166 (126–200), 3·4%	128 (103–158), 2·6%	140 (81–194), 2·9%	129 (76–223), 2·7%	80 (29–151), 1·6%	35 (20–49), 0·7%	98 (62–140), 2·0%	440 (391–491), 9·1%
	Odisha	6220 (5504–6983), 12·4%	3826 (3192–4343), 7·6%	1777 (1390–2153), 3·5%	1411 (1073–1713), 2·8%	1047 (850–1288), 2·1%	855 (604–1070), 1·7%	827 (504–1294), 1·6%	354 (130–670), 0·7%	214 (151–267), 0·4%	233 (180–274), 0·5%	3599 (3254–3990), 7·1%
	Other states†	130 (111–154), 10·2%	69 (50–85), 5·4%	53 (36–71),4·2%	54 (35–71),4·2%	<30, 1·7%	<30, 2·0%	<30, 1·4%	<30, 1·2%	<30, 0·5%	<30, 0·5%	76 (66–86), 6·0%
	Punjab	1626 (1405–1917), 14·2%	845 (613–1052), 7·4%	676 (459–905), 5·9%	687 (436–905), 6·0%	273 (221–337), 2·4%	319 (179–451), 2·8%	244 (152–373), 2·1%	<30, 0·2%	80 (45–113), 0·7%	61 (39–66), 0·5%	942 (832–1070), 8·2%
	Rajasthan	11 977 (10 658–13 482), 12·8%	7274 (6002–8301), 7·8%	3535 (2718–4310), 3·8%	2866 (2156–3498), 3·1%	2014 (1645–2479), 2·2%	1808 (1225–2324), 1·9%	1642 (1014–2510), 1·8%	66 (24–124), 0·1%	452 (306–581), 0·5%	506 (376–595), 0·5%	6941 (6294–7682), 7·4%
	Tamil Nadu	1928 (1658–2285), 8·1%	989 (705–1240), 4·1%	825 (555–1111), 3·5%	854 (535–1134), 3·6%	324 (262–400), 1·4%	403 (215–580), 1·7%	292 (183–449), 1·2%	<30, 0·1%	101 (54–145), 0·4%	84 (59–94), 0·4%	1117 (982–1277), 4·7%
	Uttar Pradesh	43 615 (39 506–47 488),13·4%	35 381 (32 597–38 287), 10·9%	6761 (5343–7779), 2·1%	3588 (2495–4522), 1·1%	7347 (5893–9190), 2·3%	6170 (4293–7795), 1·9%	7234 (4336–11 712), 2·2%	446 (163–845), 0·1%	1543 (1073–1949), 0·5%	543 (442–594), 0·2%	25 223 (22 542–28 056), 7·7%
	Uttarakhand	899 (798–1017), 12·9%	541 (441–623), 7·8%	271 (207–332), 3·9%	223 (166–272), 3·2%	151 (124–186), 2·2%	158 (96–215), 2·3%	129 (82–195), 1·8%	<30, 0·1%	40 (24–54), 0·6%	<30, 0·3%	521 (471–579), 7·5%
	West Bengal	5884 (5184–6680), 11·7%	3615 (2981–4125), 7·2%	1695 (1324–2056), 3·4%	1352 (1028–1642), 2·7%	989 (802–1213), 2·0%	1067 (629–1461), 2·1%	785 (478–1223), 1·6%	465 (171–882), 0·9%	267 (157–365), 0·5%	260 (189–317), 0·5%	3411 (3059–3792), 6·8%

Data are n (95% uncertainty interval), % of total under-5 deaths.

* Arunachal Pradesh, Manipur, Mizoram, Nagaland, Sikkim, and Tripura.

† Andaman and Nicobar Islands, Chandigarh, Dadra and Nagar Haveli, Daman and Diu, Lakshadweep, and Puducherry.

Cause-of-death distributions also varied across regions (Table 2, Table 3; appendix p 16). The two leading causes of death were preterm birth complications and pneumonia in all regions except in the south region (the region with the lowest under-5 mortality rate), where preterm birth complications and congenital abnormalities were the most prominent. Diarrhoea was the third leading cause in the northeast region, which had the highest under-5 mortality rate.

EAGA states (*vs* non-EAGA states) had a lower proportion of neonatal deaths (56·0% *vs* 63·2%), but higher proportions of preterm birth complications (28·1% *vs* 25·7%) and diarrhoea (10·6% *vs* 6·3%) as causes of death (appendix p 17). The distribution of the remaining causes of deaths was similar between the two groups.

**Figure 3:**
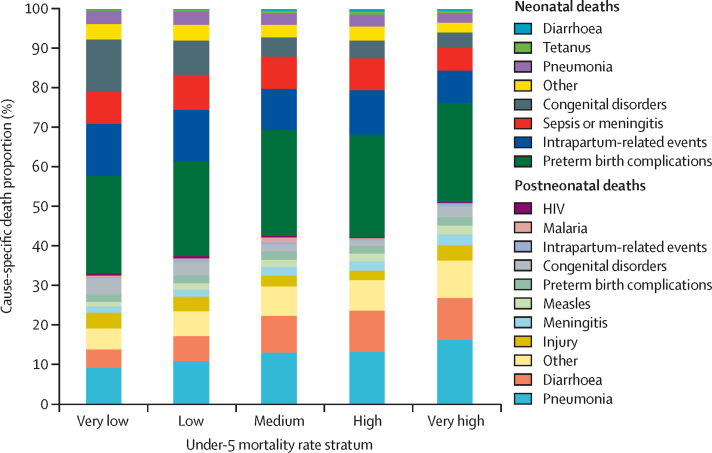
Distribution of causes of death by under-5 mortality rate stratum in 2015 35 states were grouped into five mortality strata on the basis of under-5 mortality rate in 2015: very low (≤25 deaths per 1000 livebirths; 12 states, 0·082 million under-5 deaths); low (>25–45 deaths per 1000 livebirths; 12 states, 0·250 million deaths); medium (>45–55 deaths per 1000 livebirths; six states, 0·314 million under-5 deaths); high (>55–65 deaths per 1000 livebirths; three states, 0·380 million under-5 deaths); and very high (>65 deaths per 1000 livebirths; two states, 0·175 million under-5 deaths). See appendix (p 15) for groupings.

Cause-of-death distribution also varied by stratum of under-5 mortality rate (figure 3). In states with a very low under-5 mortality rate (where the SDG under-5 mortality rate target of ≤25 deaths per 1000 livebirths has already been achieved),^13^ the three leading causes of death were all non-communicable: preterm birth complications (26·4% of under-5 deaths), congenital abnormalities (17·1%), and intrapartum-related events (13·6%). By contrast, infectious diseases were more important in states with a very high under-5 mortality rate (>65 deaths per 1000 livebirths), with the three leading causes being preterm birth complications (27·4%), pneumonia (18·7%), and diarrhoea (11·2%).

Uttar Pradesh and Bihar had the largest numbers of under-5 deaths, contributing 39·5% of the national total (table 1). The cause-of-death distributions in these two states were similar. Among neonates, preterm birth complications (25·9% of under-5 deaths in Uttar Pradesh, and 24·4% in Bihar) and intrapartum-related events (11·3% and 11·0%) were the most common causes of death, and pneumonia (13·4% and 14·0%) and diarrhoea (10·9% and 10·8%) were the leading causes among children aged 1–59 months in these two states. Neonatal deaths accounted for higher proportions of under-5 deaths in states with low mortality rates, such as Andhra Pradesh (63·3%) and Delhi (66·7%), than in Uttar Pradesh (57·6%) and Bihar (55·9%). Congenital abnormalities were among the top three leading causes of under-5 mortality in Andhra Pradesh and Delhi (appendix p 18).

Between 2000 and 2015, the number of livebirths decreased from 27·797 to 25·121 million in India,^19^ while the number of under-5 deaths decreased from 2·516 to 1·201 million, representing 32·7% of the global reduction in under-5 deaths.^1^ The under-5 mortality rate in India decreased from 90·5 to 47·8 per 1000 livebirths during this period (a 47·2% reduction). The proportion of neonatal deaths (among under-5 deaths) increased from 49·8% to 57·9% (appendix p 19).

Child survival disparities, measured by the ratio of the highest regional under-5 mortality rate (in the northeast region) to the lowest (south region), increased from 1·4 to 2·1 in 2000–15. This increase appeared to accelerate after 2005 (appendix p 20). The northeast region had the slowest decline in under-5 mortality rate, with an annual rate of reduction of 2·2% compared with at least 4·0% in other regions in this period.

Reductions in under-5 mortality rate also varied greatly across states, from 62·6% in Tamil Nadu to 26·2% in Assam. Of the five states with the highest under-5 mortality rate in 2000, four (Madhya Pradesh, Odisha, Uttar Pradesh, and Rajasthan) reduced their under-5 mortality rate by nearly half (46·6–49·3%). The exception was Assam, which had the smallest reduction in under-5 mortality rate during 2000–15, and had the highest under-5 mortality rate in 2015. When the reduction was annualised, ten of the 25 major states had annual rate of reduction in under-5 mortality rate greater than 4·4%. Most states (80%) had much slower declines in neonatal mortality rate than those among children aged 1–59 months. Uttar Pradesh, for example, had one of the highest annual rates of reduction among children aged 1–59 months (6·1%), but one of the lowest annual rates of reduction in neonatal mortality rate (2·9%; appendix pp 21–22).

Cause-specific mortality proportions changed gradually at the national level during 2000–15 (appendix pp 23–24). The three leading causes of death among neonates (preterm birth complications, intrapartum-related events, and sepsis or meningitis) were the same in 2000 and in 2015. Preterm birth complications were consistently the leading cause of death among neonates, with a cause-specific mortality rate that decreased slowly over time (annual rate of reduction 1·1%). The rate of mortality due to intrapartum-related events fell by more than 50% between 2000 and 2015, with an annual rate of reduction of 5·2%. The annual rate of reduction in neonatal cause-specific mortality was highest for tetanus (14·8%), while neonatal congenital abnormalities increased slightly (annual rate of reduction −0·1%; appendix p 25).

Among children aged 1–59 months, the three leading causes of death in 2000 were all related to infectious diseases: pneumonia, diarrhoea (including all cases), and measles. During 2000–15, the measles-specific mortality rate declined rapidly and, by 2015, injuries had overtaken as the third leading cause of death in this age group (appendix p 24). Annual rates of reduction in this age group ranged from 2·0% (for congenital abnormalities) to 7·1% (for measles; appendix p 25).

Disparities in annual rates of reduction in 2000–15 were observed across causes and across states (appendix pp 26–27). Generally, annual rate of reductions were higher for infectious causes than for non-communicable causes across states.

**Figure 4:**
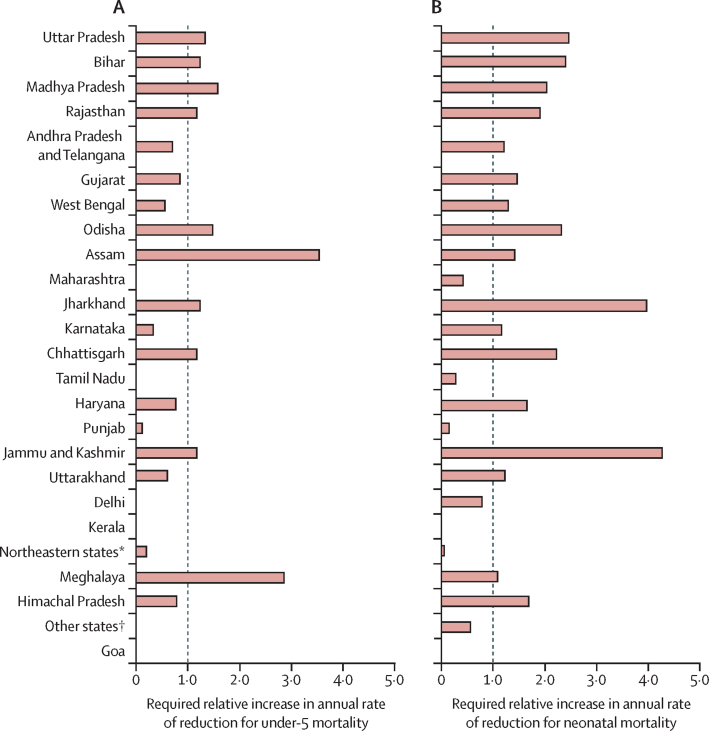
Relative acceleration required by each state to meet Sustainable Development Goal targets on under-5 and neonatal mortality by 2030 Data are the required annual rate of reduction (in 2015–30) divided by the annual rate of reduction achieved in 2000–15. States with a required relative increase of less than 1·0 are on track to meet the target by 2030. States with missing values have already met the target. States are ordered by number of under-5 deaths in 2015, with the highest at the top and lowest at the bottom. *Arunachal Pradesh, Manipur, Mizoram, Nagaland, Sikkim, and Tripura. †Andaman and Nicobar Islands, Chandigarh, Dadra and Nagar Haveli, Daman and Diu, Lakshadweep, and Puducherry.

Although a few states have already achieved and some others are on track to achieve the SDG child survival targets, the remaining states, and India as a whole, will need to accelerate progress substantially. To achieve the SDG under-5 mortality rate target by 2030, ten of the 25 major states need to accelerate progress in terms of their annual rate of reduction during 2015–30 (figure 4), including the four states (Uttar Pradesh, Bihar, Madhya Pradesh, and Rajasthan) with the largest numbers of under-5 deaths. To achieve the neonatal mortality rate targets, 17 of the 25 major states need to accelerate progress (figure 4).

## Discussion

In 2015, India had the largest number of under-5 deaths globally, and almost 60% of these deaths occurred in the neonatal period. Compared with estimates from the National Family Health Survey 4, our estimated under-5 mortality rate and neonatal mortality rate were similar in the 2010–15 period, but showed a faster decline since 2000, both nationally and in major states.^3^ The leading causes of death were preterm birth complications, pneumonia, and intrapartum-related events. Neonatal sepsis or meningitis, diarrhoea, and injuries were also important causes. Under-5 mortality rate almost halved in India between 2000 and 2015, largely due to fast declines in causes that could be potentially addressed by vaccines. Although mortality due to intrapartum-related events declined substantially in 2000–15, mortality due to preterm birth complications showed little change, suggesting improvements in labour and delivery practices but not in care of neonates born preterm. For example, programmes to increase institutional births, ambulance support for referral, and caesarean section in areas where these facilities were low^3^ might have contributed to the decline in intrapartum-related events. However, the establishment of several hundred district neonatal intensive care units^31^ does not appear to have had a noticeable effect on mortality due to preterm birth complications.

Geographical and socioeconomic disparities in child survival exist throughout India. Regional disparities worsened during 2000–15, especially after 2005. Pneumonia and diarrhoea were among the top three leading causes in the northeast region, which had the highest under-5 mortality rate. Preterm birth complications were particularly important in the EAGA states. Disparities were also present across age groups and causes. In the majority of the states, reductions in under-5 mortality rate were driven by reductions in deaths among children aged 1–59 months, whereas most states had a much slower decline in neonatal mortality rate. The relative importance of preterm birth complications increased over the 2000–15 period in states with large numbers of under-5 deaths. Diarrhoea had a faster decline than many other causes across almost all states. However, the decline was slower in states with high versus those with low under-5 mortality rates.

National efforts have been made to improve child survival in 2000–15 in India. The National Rural Health Mission was implemented in 2005 to increase maternal and child health services in rural areas, strategically focused in EAGA states and the northeast region, which might have contributed to the improvement in child survival and mitigated regional disparities.^32^, ^33^, ^34^, ^35^, ^36^, ^37^, ^38^ As a reference, the ratio of the highest regional under-5 mortality rate (in the northeast region) to the lowest (in the south region) in India was 2 in 2015, compared with more than 4 in the highest and lowest in China.^39^ As the universal health coverage agenda is further advanced in India, child survival equity can hopefully be better addressed.^33^

Progress in India is crucial to improving child survival globally. The leading causes of preterm birth complications, pneumonia, intrapartum-related events, neonatal sepsis or meningitis, diarrhoea, and injuries should receive more attention from child survival policy makers and programmes. In this study, we considered the effect of the Hib vaccine on pneumonia and meningitis mortality. The newly introduced pneumococcal conjugate vaccine^40^ and second-dose measles vaccine^41^ are expected to further reduce mortality due to pneumonia and measles. Efforts to increase vaccination coverage, including Mission Indradhanush^10^, ^42^ and a measles–rubella vaccination campaign, launched by the government of India in 2017, might help to further reduce the number of deaths due to infectious causes.

Preterm birth complications were the leading cause of under-5 deaths in India in 2000–15, and the rate of mortality from this cause saw little change during this period, especially in EAGA states. Cost-effective interventions, such as kangaroo care, thermal control, breastfeeding support, and basic care for infections^43^ and breathing difficulties, could be considered.^44^, ^45^, ^46^ Efforts in integrated newborn care packages, such as integrated management of neonatal and childhood illnesses,^36^, ^47^ could further improve neonatal health outcomes. However, evaluation of health initiatives shows varying programme governance and accountability across states. Therefore, high and consistent commitment from local government is crucial to ensure that programmes work as intended.^35^, ^48^

Diarrhoea remains an important cause of death, particularly in states with high under-5 mortality rates and low socioeconomic status. However, improvement of diarrhoea survival has never been more promising.^49^ Access to diarrhoea treatments, such as oral rehydration solution and zinc supplements, has significantly increased in the past decade^3^, ^4^ through the engagement of accredited social health activist workers in community-based health service provision.^50^, ^51^, ^52^ Nationwide campaigns, such as the Intensified Diarrhoea Control Fortnight, was launched in 2014 to create mass awareness of diarrhoea treatments.^53^ With the scale-up of these interventions, diarrhoea survival could be further improved in Indian states with high mortality.

The differences in numbers of cause-specific deaths between our study (MCEE), MDS, and GBD were largely due to 17% fewer all-cause under-5 deaths and 8% fewer livebirths estimated by the UN Inter-agency Group for Child Mortality Estimation than were estimated by GBD (appendix p 28).^1^, ^19^, ^29^ MCEE and GBD estimates for prematurity were not directly comparable to those from the MDS. The two physicians who reviewed each MDS record were not asked to distinguish between deaths due to disorders related to slow fetal growth and fetal malnutrition (P05) and those due to disorders related to short gestation and low birthweight (P07). More careful examination of the MDS data revealed that 56% of neonatal deaths coded as prematurity or low birthweight were reported as full-term low birthweight. MDS is investigating the feasibility of requiring physicians to distinguish between P05 and P07 in the future. For deaths at 1–59 months, where differences in cause-of-death classification were minor, MCEE estimated slightly more deaths due to pneumonia and diarrhoea and fewer due to injury and malaria (appendix p 30) than were estimated by MDS and GBD. State-level trends in all-cause mortality rates, mortality rates for neonatal infections, birth asphyxia or trauma, pneumonia, and diarrhoea were similar between MDS and MCEE, with the exception of some smaller and new states with less information reported (eg, Assam, Jharkhand, and Chhattisgarh; appendix pp 31–37).

Each of the approaches used to estimate state-level mortality rates in this study and in MDS had strengths and limitations. MCEE estimates were based on standardised procedures, making the estimates internationally comparable, but they might be less capable of reflecting short-term changes. By contrast, MDS estimates were based on annual data, which show more fluctuations for smaller causes (data not shown). Such fluctuations could reflect both real changes and data quality issues. Although representative and timely data from a high-quality vital registration system would be the ideal source of data, we believe in the value of both estimation approaches, which serve different audiences and purposes. We intended to compare our approach with the GBD state-level estimates, but were unable to do so because GBD estimates are only available through the visualisation tool, but are not yet downloadable. Improved transparency would promote understanding, comparison, and enhancement of global health estimates.^30^

The present study had some limitations. First, many states in the northeast region are small states with little data available from the sample registration system. To fill these data gaps, for example on neonatal mortality rate, we chose to borrow information from nearby states (in this case, Assam and West Bengal). In Assam, the proportion of under-5 deaths that were neonatal was 37·3%. States in the northeast for which information was borrowed from Assam had a large share of the regional under-5 deaths, and the relatively low proportion of neonatal deaths in Assam might have led to an artificially low proportion of neonatal deaths in the entire northeast region. This borrowing of data would also affect cause-specific estimates because cause-specific mortality fractions were applied to all-cause deaths among neonates and older children separately. Consequently, although preterm birth complications were the leading cause among neonates, they were not the leading cause among all children under 5 years of age in the northeast region. This limitation might also explain why, despite a high under-5 mortality rate, preterm birth complications were not the leading cause in the northeast region but were in the other regions.

A second limitation was that, although the modelling strategies used for neonates and older children were similar, the two age groups relied on different input data: neonatal data were taken from verbal autopsy studies from all high-mortality countries, whereas data for older children relied on Indian subnational verbal autopsy studies. Such a difference was justified because data availability varies by age group. The number of studies reporting Indian subnational causes of death among neonates was too small to derive stable estimates. Additionally, many of the India subnational studies had missing data for important causes such as pneumonia, thereby further reducing the reliability of estimates based only on Indian studies. When comparing the input data from India versus other high-mortality countries, we found no substantial differences in the cause-of-death distributions among neonates (appendix p 7). Thus, we chose to use all high-mortality studies as inputs for the neonatal modelling.

Finally, our uncertainty calculations did not consider all sources of errors. For example, although we adopted the exponential growth assumption when interpolating for annual population estimates, the likely errors (albeit small) were not explicitly propagated in our uncertainty estimates. Similarly, borrowing estimates (eg, neonatal mortality rate) from another state might have incurred errors, and such errors were also not explicitly accounted for in our uncertainty estimation. To do so, we would have had to make assumptions about the distribution of such errors, which itself cannot be empirically tested.

In summary, our estimates are based on the most comprehensive set of high-quality, subnational, verbal autopsy studies to date. This study provides reliable, albeit conservative, time trends on all-cause and cause-specific under-5 mortality at the national and subnational levels in India with transparency. Future work is needed to further increase the resolution of estimates on all-cause estimates and particularly cause-specific estimates (eg, at district level). The demand for this information is driven by decentralised decision making and child survival programme efforts, such as the scaling-up of pneumococcal conjugate vaccine and the continuation of Mission Indradhanush at district level. Among older children, as vaccine coverage data were used as model inputs and in post-hoc adjustment, these estimates should not be used to infer causality between vaccine and mortality decline.

The evidence base for child mortality and causes of death can be improved through a combination of more and better data collection. Standard verbal autopsy instruments and cause-ascertaining methods should be implemented. Innovative data collection, such as minimally invasive tissue sampling^54^ or Countrywide Mortality Surveillance for Action,^55^ could be considered for better measurement of cause of death and potentially for more efficient and sustainable data acquisition. Advanced modelling approaches are essential to derive more reliable estimates. The direct and indirect effects of existing and newly scaled-up vaccines on infectious diseases should be routinely considered. National and subnational civil registration and vital statistics systems can be strengthened through in-country capacity building so that data on cause-specific mortality are routinely collected, promptly published, and publicly released using standard, transparent, and reproducible methods.^5^ At the subnational level, building data capacity within local health systems, combined with collection of better and more timely data, and advances in estimation techniques for small areas,^56^ could lead to more precise estimates of child mortality and causes of death, which will, in turn, inform more effective child survival policy planning and programme evaluation in the SDG era.

For access to **study data on the MCEE website** see https://www.jhsph.edu/research/centers-and-institutes/institute-for-international-programs/current-projects/maternal-child-epidemiology-estimation/maternal-newborn-and-child-cause-of-death/index.html

For more on **Spectrum** see https://www.avenirhealth.org/software-spectrum.php

## Data sharing

Additional details of the input data, estimation methodology (including statistical codes), and estimates are publicly available through the MCEE website.
