# Supplementary material for: National, regional, and state-level all-cause and cause-specific under-5 mortality in India in 2000–15: a systematic analysis with implications for the Sustainable Development Goals
Source: Lancet Glob Health. 2019 May 13;7(6):e721–34. doi: 10.1016/S2214-109X(19)30080-4 (PMC6527517; doi:10.1016/S2214-109X(19)30080-4)
Supplement: Supplementary appendix [file mmc1.pdf]

THE LANCET
Global Health

Supplementary appendix

This appendix formed part of the original submission and has been peer reviewed. We post it as supplied by the authors.

Supplement to: Liu L, Chu Y, Oza S, et al. National, regional, and state-level all-cause and cause-specific under-5 mortality in India in 2000–15: a systematic analysis with implications for the Sustainable Development Goals. *Lancet Glob Health* 2019; **7:** e721–34.

National, regional and state all-cause and cause-specific under-five mortality in India in 2000-2015: implications for the Sustainable Development Goals

Li Liu, PhD*[1,2] Yue Chu, MSPH*[2] Shefali Oza, PhD [3] Dan Hogan, PhD [4] Jamie Perin, PhD[2] Diego G Bassani, PhD [5,6] Usha Ram, PhD[7#] Shaza A. Fadel, PhD [5] Arvind Pandey, PhD [8] Neeraj Dhingra, MD [8] Damodar Sahu, PhD [8] Pradeep Kumar, PGDHM[9] Richard Cibulskis, PhD [10] Brian Wahl, PhD [11] Anita Shet, MD [11] Colin Mathers, PhD [4] Joy Lawn, PhD [3#] Prabhat Jha, DPhil [5#] Rakesh Kumar, MD[12] Robert E. Black, MD [2#] and Simon Cousens, MA[3#]

* contributed equally
# indicates full professor

[1]Department of Population Family and Reproductive Health, Johns Hopkins Bloomberg School of Public Health, Baltimore, Maryland
[2]The Institute for International Programs, Department of International Health, Johns Hopkins Bloomberg School of Public Health, Baltimore, Maryland
[3]Department of Infectious Disease Epidemiology, London School of Hygiene and Tropical Medicine, London, UK
[4]Health Metrics and Measurement Cluster, World Health Organization, Geneva, Switzerland
[5]Dalla Lana School of Public Health, University of Toronto, Toronto, Canada
[6]Centre for Global Child Health, The Hospital for Sick Children, Department of Paediatrics, University of Toronto, Canada
[7]Department of Public Health & Mortality Studies, International Institute for Population Sciences, Mumbai, India
[8]National Institute of Medical Statistics (Indian Council of Medical Research), New Delhi, India
[9]National AIDS Control Organization, New Delhi, India
[10]Global Malaria Programme, World Health Organization, Geneva, Switzerland
[11]International Vaccine Access Center, Department of International Health, Johns Hopkins Bloomberg School of Public Health, Baltimore, Maryland
[12]United Nations Development Programme, India

Correspondence to: Dr. Li Liu, Department of Population, Family and Reproductive Health, and the Institute for International Programs, Department of International Health, Johns Hopkins Bloomberg School of Public Health, 615 N Wolfe Street, Baltimore, MD 21205, USA
lliu26@jhu.edu

Webappendices

Webappendix 1. Details on estimating state live births, under-five all-cause mortality rates and number of deaths, India, 2000-2015

Webappendix 2. Details on estimating national, regional and state cause-specific mortality fractions in 2000-2015

Webappendix 3. Procedures for the estimation of deaths by cause in children younger than 5 years by state in India

Webappendix 4. State grouping

Webappendix 5. GATHER checklist

Webappendix 6. Under-five cause of death distribution by region in India in 2015

Webappendix 7. Under-five cause of death distribution in EAGA and non-EAG states in India in 2015

Webappendix 8. Under-five cause of death distribution in selected states in India in 2015

Webappendix 9. U5MR and NMR at the national and regional levels in India in 2000-2015

Webappendix 10. Ratio of U5MR between the Northeast and South Regions in India in 2000-2015

Webappendix 11. Annual rate of reduction of U5MR, NMR, mortality rate among 1-59 month olds by state in India in 2000-2015

Webappendix 12. National trends in cause-specific mortality fractions among a) neonates and b) children aged 1-59 months in India in 2000-2015

Webappendix 13. Annual rate of reduction in cause-specific mortality rates among neonates and children aged 1-59 months at national and regional level in India between 2000 and 2015

Webappendix 14. Annual rate of reduction of cause-specific mortality rates due to leading under-five causes by state in India between 2000 and 2015

Webappendix 15. Comparisons of all-cause mortality and live births estimates for year 2015 between the UN Inter-agency Group for Child Mortality Estimation and the Global Burden of Disease Study

Webappendix 16. Comparison between MCEE, MDS and GBD of cause-specific mortality fractions of a) neonatal deaths and b) deaths among those aged 1-59 months in India in 2015

Webappendix 17. Comparisons between MCEE and MDS of state all-cause neonatal, 1-59-month deaths, and livebirths estimates in India in 2000-2015

Webappendix 18. Comparisons of trends in mortality rates due to a) the three leading causes of neonatal deaths and b) pneumonia and diarrhea in big states in India in 2000-2015

Webappendix 1. Details on estimating state live births, under-five all-cause mortality rates and number of deaths, India, 2000-2015

1.1. Population

National and state population estimates in 1991, 2001 and 2011 were taken from the corresponding censuses.[1] Annual population estimates in 1992-2000 and 2002-2010 were interpolated using annual exponential growth rates estimated between two consecutive censuses of 1991 and 2001, and 2001 and 2011, respectively. Population estimates in 2012-2015 were extrapolated using exponential annual growth rates based on 2001 and 2011 censuses. The 1991 Census was not held in Jammu & Kashmir. We interpolated using data for Jammu & Kashmir from the 1981 and 2001 censuses. Population of Manipur included that of Mao Maram, Paomata and Purul sub-divisions of Senapati district in 2001.

1.2. Crude birth rates (CBR), infant mortality rates (IMR), and neonatal mortality rate (NMR)

CBR, IMR and NMR were taken from the Sample Registration System (SRS).[2-4] When CBR/IMR/NMR was unavailable, the following was done:

- Chhattisgarh, Jharkhand and Uttarakhand were each carved out from Madhya Pradesh, Bihar, and Uttar Pradesh in 2000/2001, respectively. The SRS did not publish CBR in 1999-2000 for Chhattisgarh, and in 1990-1999 for Jharkhand and Uttarakhand. For these missing years, CBR was estimated using the ratio of CBR of the daughter state to that of her mother state in 2001. For example, to estimate CBR for years before Chhattisgarh came into existence, the ratio of CBR of Chhattisgarh (daughter state) to Madhya Pradesh (mother state) in 2001 was computed and applied to obtain the missing CBR.
- CBR/IMR were obtained for Dadra and Nagar Haveli in 1990-1994 by applying the ratio of CBR/IMR between Dadra and Nagar Haveli and Gujarat in 1995 to CBR/IMR for Gujarat in 1990-1994. Similarly, CBR for Mizoram in 1990-2004 was obtained by applying the ratio of CBR between Mizoram and Assam to CBR for Assam in 2004.
- CBR for Jammu & Kashmir in 1991-1997 was interpolated assuming linear annual change between 1990 and 1998. Similarly, CBR for Nagaland in 1995-2004 was interpolated assuming linear annual change between 1994 and 2005.
- The SRS did not publish IMR in 1999-2003 for Chhattisgarh and Jharkhand, and in 1990-2006 for Uttarakhand. For these missing years IMR was estimated using the ratio of the IMR of the daughter state to that of her mother state observed in 2004 as done for CBR.
- The following interpolation was done to fill missing IMR using data available right before and after the missing years: Andaman and Nicobar Islands in 1991-1996, Chandigarh in 1990-1992, Nagaland in 1991-2004, Delhi in 1991-2006, Himachal Pradesh in 1991-2006, Daman and Diu in 1991-2008. That was, for example, IMR for Andaman and Nicobar Islands in 1991-1996 was interpolated assuming linear annual change between 1990 and 1997.

- IMR for Jammu & Kashmir in 1991-1997 and 1999-2000 was estimated by applying the ratio of IMR between Jammu & Kashmir and Himachal Pradesh in 1998.
- IMR for Mizoram in 1990 was assumed to be the same as that of Assam in the same year. The IMR for Mizoram in 1991-2007 was obtained assuming linear annual change between 1990 and 2008.
- IMR for Goa in 1994 was computed as the average of IMR in 1993 and 1995.
- For Lakshadweep, IMR in 1990 was assumed to be the same as that of 1991. In 1992-1993, IMR was interpolated assuming linear annual change between 1991 and 1994. In 1996, IMR was taken as the average of 1994 and 1995.
- NMR in 1990-2014 was taken from SRS for India and 20 big states (Andhra Pradesh, Assam, Bihar, Gujarat, Haryana, Karnataka, Kerala, Madhya Pradesh, Maharashtra, Odisha, Punjab, Rajasthan, Tamil Nadu, Uttar Pradesh, and West Bengal). For the remaining 15 states, NMR was estimated by applying the ratio of IMR between the state and that of its reference state for the same years to IMR of the reference state. The reference states are listed in the Webappendix Table 1 below.

Webappendix Table 1. States with missing NMR and their reference states

**State/Union Territory**	**Reference state**
Andaman and Nicobar Islands	West Bengal
Arunachal Pradesh	Assam
Chandigarh	Haryana
Chhattisgarh	Madhya Pradesh
Dadra and Nagar Haveli	Gujarat
Daman and Div	Gujarat
Delhi	Haryana
Goa	Maharashtra
Himachal Pradesh	Punjab
Jammu Kashmir	Himachal Pradesh
Jharkhand	Bihar
Lakshadweep	Kerala
Manipur	Assam
Meghalaya	Assam
Mizoram	Assam
Nagaland	Assam
Puducherry	Tamil Nadu
Sikkim	West Bengal
Tripura	West Bengal
Uttarakhand	Uttar Pradesh

1.3. Under-five mortality rate (U5MR)

<u>India and 20 big states</u>

For 2008-2013, U5MR for the 20 big states was taken from the SRS (Statement no. 54). [3] Before 2008, SRS did not publish state U5MR. However, it did publish IMR (${}_1q_0$) and age specific death rates (ASDR) at ages 1-4 years. The mortality rates at ages 1-4 years were then converted into probability of dying using Greville's method.[5] Specifically:

$${}_4q_1 = \frac{ASDR_{1-4}}{\frac{1}{4} + ASDR_{1-4} * [0.5 + (\frac{4}{12}) * (ASDR_{1-4} - 0.095)]}$$

$${}_5q_0 = 1 - ((1 - {}_4q_1) * (1 - {}_1q_0))$$

Where 1q0 is the probability of dying between births and age one year or IMR. 1-4q1=4p1, which is the probability of surviving between ages one to five years. 1-1q0=1p0, which is the probability of surviving between birth to age one year. 4p1*1p0=5p0, which gives the probability of surviving between birth to age five years. 1-5p0=5q0, which is the probability of dying between birth and age five years or U5MR.

<u>The remaining 15 small states and union territories</u>
U5MR was estimated applying the ratio of IMR between the state and that of its reference state for the same years. The reference states are listed in the Webappendix Table 1 above.

For 2014 for India and states for which SRS did not published the estimates and for 2015, CBR and mortality rates were estimated using annual change between 2010/11 and 2013/14 as applicable.

1.4. Share of state/union territory population, live births and age-specific deaths

Once rates for each year/state were estimated, the year-state specific absolute numbers of live births and deaths were obtained and adjusted (by no more than 3%) to ensure that the sum of all states matched that of the national total for each year. Dividing state numbers by the India national total gave the share of state events.

To ensure international comparability, we further adjusted India national and state estimates to be consistent with estimates produced by the United Nations (UN). The annual population of India was taken from the World Population Prospects. The total number of live births was computed using annual birth rates published by the UN. [6] The NMR, IMR and U5MR were taken from the UN Inter-agency Group on Child Mortality Estimation. [7] Using the relative share of state events out of the national total as published by the UN, we obtained state specific numbers of population, live births, and neonatal/infant/under-five deaths. Subtracting neonatal deaths from under-five deaths gave deaths at the ages of 1 to 59 months.

Webappendix 2. Details on estimating national, regional and state cause-specific mortality fractions (CSMFs) in 2000-2015

2.1. Rationale for using verbal autopsy (VA) studies from all high mortality countries to model CSMFs among neonates

We chose to use VA studies from all high mortality countries rather than only using India subnational VA studies for three reasons. First, excluding data from all other high-mortality countries resulted in a smaller input neonatal dataset of 18 studies including about 14,000 deaths. Such a small dataset was more prone to estimation instability. Second, the number of India subnational studies missing data for certain causes required in the multinomial model was high. In particular, only about 40% of the Indian subnational study data points in the neonatal database included pneumonia as a separate cause, resulting in only 140 recorded pneumonia deaths. These numbers were too small to produce stable estimates for pneumonia. Finally, when comparing input data from India versus other high-mortality countries, there did not appear to be any substantial differences in the cause-of-death distributions among neonates (Webappendix Figure 2).

Webappendix Figure 1. CSMFs in VA studies from high mortality countries versus those from India subnationally, neonates

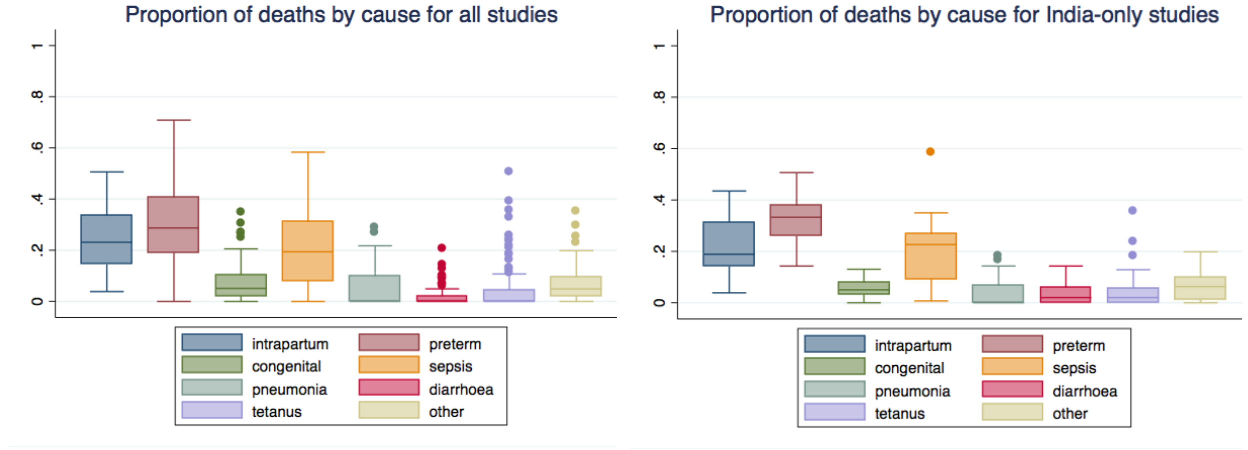

2.2. Rationale for using subnational VA studies within India to model CSMFs among 1-59-month olds

For the estimation of CSMFs among older children, we developed a subnational VA based multi-cause model (VAMCM) to better account for cause of death variations at state level in India. We chose to use within India subnational VA studies because they showed different cause-of-death distribution compared to that from all high mortality countries (Webappendix Figure 2). Specifically, India had higher medians of the distribution of CSMFs of diarrhea and pneumonia, yet lower medians of intrapartum related events/preterm birth complications/congenital abnormalities, compared to the high mortality countries. We also conducted a sensitivity analysis predicting India subnational estimates using VA studies from all high mortality countries and the results were different

and less plausible (now shown). We considered it strength to use subnational VA studies within India among older children.

Webappendix Figure 2. CSMFs in VA studies from high mortality countries versus those from India subnationally, children aged 1-59 months

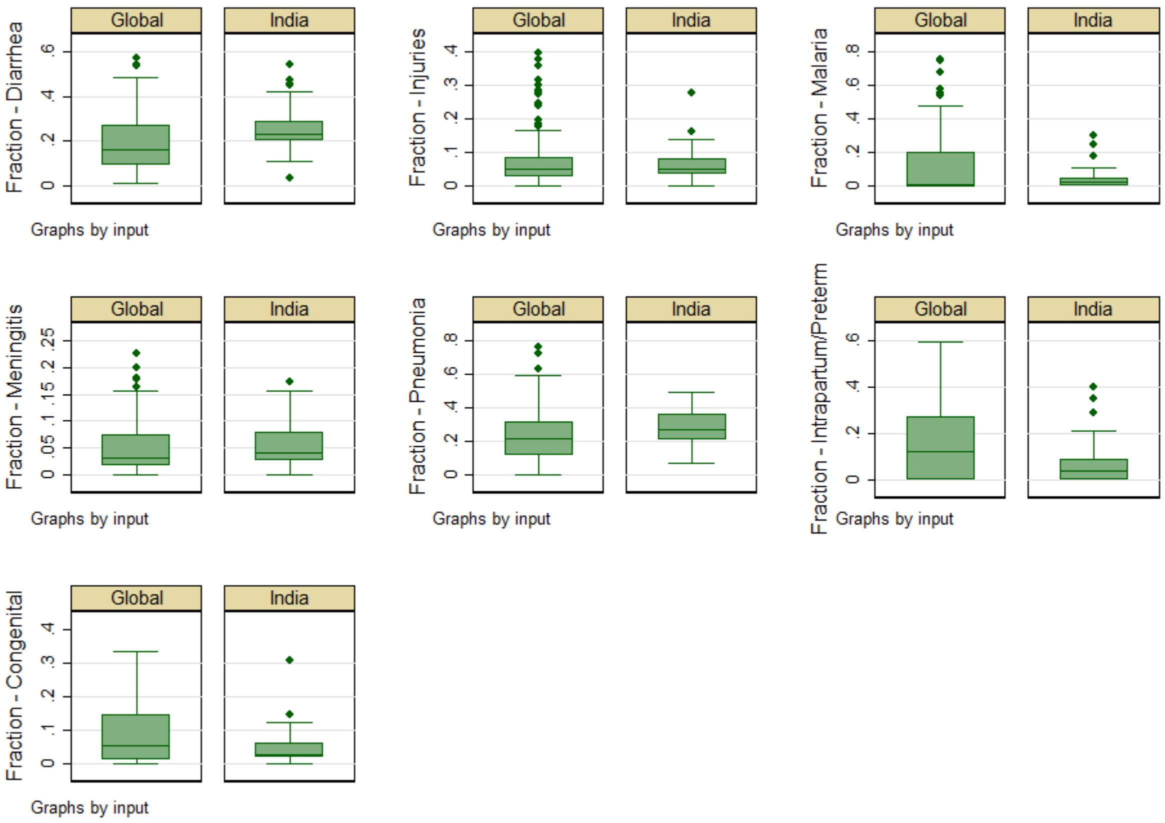

Note: “Global” indicates CSMFs from VA studies from all high mortality countries. “India” indicates CSMFs from subnational VA studies from India .

2.3. Deaths due to AIDS

Deaths due to HIV/AIDS were derived for all states and union territories using Spectrum.[8] For each state, historical surveillance data for women visiting antenatal clinics and for high risk groups (female sex workers, men who have sex with men, and injecting drug users) were used to model HIV prevalence and incidence in the respective population. Using programmatic data on prevention of mother-to-child transmission and antiretroviral treatment for adults and children and modelling the progression from new HIV infection to AIDS in the presence or absence of treatment, Spectrum derived estimates of AIDS related deaths for children.

Each person infected with HIV followed a specific pattern of survival from infection to death depending upon the time when he/she started the treatment. A number of studies modelled the progression from new HIV infection to AIDS in the absence of treatment, the

progression from infection to the need for treatment according to different levels of CD4 counts, and the progression from need for treatment to AIDS death with or without treatment. Spectrum included such evidence in its modelling using a *Weibull* function that described the proportion dying by time since infection. It used a simple logic function assuming that at the time of infection, the CD4 levels were generally high, and with time it kept decreasing till it reached a level that causes death. [9]

Spectrum computed for each year separately the following quantities: (1) the number of AIDS related deaths among those who have not started any treatment since their infection, and (2) the number of AIDS related deaths among those who are on treatment for each CD4 category. To avoid inflating the estimates, the system calculated mortality among the HIV positive population for causes not related to AIDS by applying the same mortality patterns as for the non-HIV population, and subtracted it from the total estimated number of AIDS deaths. For children, in the absence of antiretroviral therapy and cotrimoxazole prophylaxis, children infected through vertical transmission progress over time to AIDS death according to a double Weibull curve. Different progression patterns were used for children infected prenatally and those infected at the ages of 0-6 months, 7-12 months, and >12 months postpartum through breastfeeding.[10-12] The estimated numbers of AIDS deaths among children were derived using this method by age, representing the duration from infection to death according to when the infection happened (during pregnancy, at birth, or during breastfeeding). The fractions of neonatal deaths out of under-five deaths due to HIV/AIDS were assumed to be the same as those at the national level at 1% as estimate by UNAIDS. [13] The uncertainty bounds were calculated using the uncertainty option for this indicator in Spectrum.

2.4. Deaths due to malaria

The numbers of deaths due to malaria in 2000-2015 at the national level and in 2000-2013 at the state level were estimated in two steps:

Step 1: Number of cases was estimated made by adjusting the number of reported malaria cases for reporting completeness and health service use. The procedure was described in the World malaria report 2008.[14,15] It combined data reported by national malaria control programmes on cases and reporting completeness with those obtained from nationally representative household surveys on health service use. The number of P. vivax malaria cases in each country was estimated by multiplying the country's reported proportion of cases that were P. vivax by the total number of estimated cases for that country.

Step 2: Number of deaths were estimated by multiplying the estimated number of malaria cases by a fixed case fatality rate.[15] A case fatality rate of 0.01%-0.40% was applied to the estimated number of *P. falciparum* cases, and a case fatality rate of 0.01%-0.06% was applied to the estimated number of *P. vivax* cases. For years 2014 and 2015, the number of deaths due to malaria for each state was calculated assuming that the state distribution of national malaria deaths was the same as that of 2013.

2.5. State net domestic product (NDP) per capita

State NDP at factor cost per capita at constant price was used as one covariate.[16] The raw NDP reported were real NDP, which were evaluated at the market prices of certain base years to account for inflation or deflation over time. NDP in 2000-2004 were reported using 1999-2000 market price as the base, while NDP in 2004-2015 were reported using 2004-2005 market price as the base. To make NDP per capita comparable across time, all raw NDP data points reported in the handbook with different base were converted to using 1999-2000 constant price as the base by applying an adjustment factor to NDP in 2005 onwards using the following equation.

$$NDP\ for\ year\ i_{1999-base} = NDP\ for\ year\ i_{2004-base} \times \frac{NDP\ for\ year\ 2004_{1999-base}}{NDP\ for\ year\ 2004_{2004-base}}$$

2.6. Study covariate missing imputation

For study covariates with missing values for certain state and years (e.g. underweight, low birth weight, insecticide treated net, and stunting), values from other states were borrowed.[17] The borrowing matrix (Webappendix Table 2) was largely consistent with that for NMR. If the values were still missing, regional or national averages were used.

Webappendix table 2. Matrix for states with missing covariate values

State with missing value	Substitute state
Andaman and Nicobar Islands	Karnataka
Arunachal Pradesh	Assam
Chandigarh	Haryana
Dadra & Nagar Haveli	Gujarat
Daman-Diu	Gujarat
Goa	Maharashtra
Lakshadweep	Kerala
Manipur	Assam
Mizoram	Assam
Nagaland	Assam
Puducherry	Tamil Nadu
Sikkim	Assam
Tripura	Assam
Uttaranchal	Uttar Pradesh

2.7. State prediction covariate database preparation

State prediction covariates were extracted from India Economic Yearbooks for state NDP per capita, and from National Family Health Survey (NFHS), Annual Health Survey (AHS) and District Level Household Survey (DLHS) for all the other covariates.[16] For covariates with missing values for certain state and years, values from other states were borrowed,[17] or regional or national averages were used. Data were then linearly interpolated for years between two empirical data points. For years before the first and after the last available surveys, estimates were extrapolated assuming a flat trend. All covariates except mortality rates and vaccine coverage were smoothed using locally weighted scatterplot smoothing to derive stable state covariates for the prediction of CSMFs. Given the population size of India and its major states, we tend to believe that the trends of child causes of deaths were rather

stable in general than bumpy, thus we hope certain level of smoothing for covariates used for prediction would help to increase stability and enhance validity.

2.8. Covariates retained in the VAMCM

Neonates		1-59 months	
Cause	Covariates	Cause	Covariates
Intrapartum	(ref.)	Pneumonia	(ref.)
Congenital	Period; SBA; U5MR; Region;	Injuries	Stunting;
Preterm	GFR; LBW; SBA; NMR; U5MR;	Measles	Measles vaccine;
Pneumonia	BCG; SBA;	Meningitis	Intc.;
Diarrhoea	LBW;	Other	Intc.;
Other	PAB; ANC; BCG; Region;	Neonatal	Age range; U5MR
Tetanus	Period; NMR; Region;	Congenital	Stunting;
Sepsis	ANC; Female literacy; Period; PAB;		

Abbreviations: ref.: as reference; ANC: antenatal care; BCG: Bacillus Calmette–Guérin vaccine coverage; GFR: gross fertility rate; Intc.: intercept; LBW: low birth weight; NMR: neonatal mortality rate; PAB: neonates protected at birth against neonatal tetanus; SBA: deliveries assisted by skilled birth attendants; U5MR: under-five mortality rate;

2.9. Post-hoc adjustment

We adjusted the impact of *Haemophilus* influenza type B (Hib) vaccine on deaths due to pneumonia and meningitis. [13] We calculated pneumonia- and meningitis-specific deaths averted by Hib vaccine use based on fractions due to vaccine coverage, vaccine effectiveness, and modeled cause-specific deaths. Then we redistributed these deaths averted due to Hib to other modeled causes pro rata.

The numbers of cause-specific deaths that could have been averted by Hib use were calculated using the following equation:

$$CSN_{averted} = CSN_{modeled} \times SSP_{serotype} \times \mathrm{C} \times \mathrm{Eff}$$

where $CSN_{averted}$ denotes the number of cause-specific number (CSN) of deaths averted with vaccine use. $CSN_{modeled}$ represents VAMCM modeled cause-specific number of deaths. $SSP_{serotype}$ is the proportion of cause-specific deaths due to the vaccine-specific serotype if no vaccine coverage, and was assumed to be 46% [18] for the effect of Hib on meningitis mortality, and 21%[19] for its effect on pneumonia mortality. *Eff* is vaccine efficacy, which was assumed to be 95%[20] for the effect of Hib effect on meningitis, and 93%[21] for the effect of Hib on pneumonia. *C* denotes vaccine coverage. Since no subnational estimates of Hib vaccine coverage were readily available, we used coverage estimates for the third dose of

diphtheria-tetanus-pertussis vaccine (DTP3) from subnational surveys as a proxy for coverage with three doses of Hib vaccine. The subnational surveys used include DLHS-3)[22], DLHS-4[23], AHS[24-26], and NFHS-4[27] 2015-2016. We prorated DTP3 coverage estimates in the year when Hib vaccine was only introduced for part of the year. Linear interpolation was used when data were missing for certain years.

2.10 Uncertainty estimates

We estimated uncertainty in model coefficients by bootstrap resampling of input data sets from all estimation components and their respective distributions with replacement. This uncertainty was propagated through to the model predictions. The 2·5 and 97·5 percentiles were taken as the lower and upper ranges of the uncertainty.

2.11 Calculation of annual rates of reduction (ARR)

We calculated annual rates of reduction (ARR) using the following equation,[28]

$$ARR = \frac{\ln(\frac{CSMR_{year_2}}{CSMR_{year_1}})}{(year_2 - year_1)} \times 100 \times (-1)$$

where ARR is annual rate of reduction between $year_1$ and $year_2$, $CSMR_{year_i}$ is cause-specific mortality rate at year i.

The benchmark ARR required to achieve MDG 4, where the national goal was to reduce under-five mortality rates by two thirds between 1990 and 2015, was calculated as follow.

$$ARR = \frac{\ln(1/3)}{(2015 - 1990)} \times 100 \times (-1) \approx 4.4$$

Webappendix 3. Procedures for the estimation of deaths by cause in children younger than 5 years by state in India

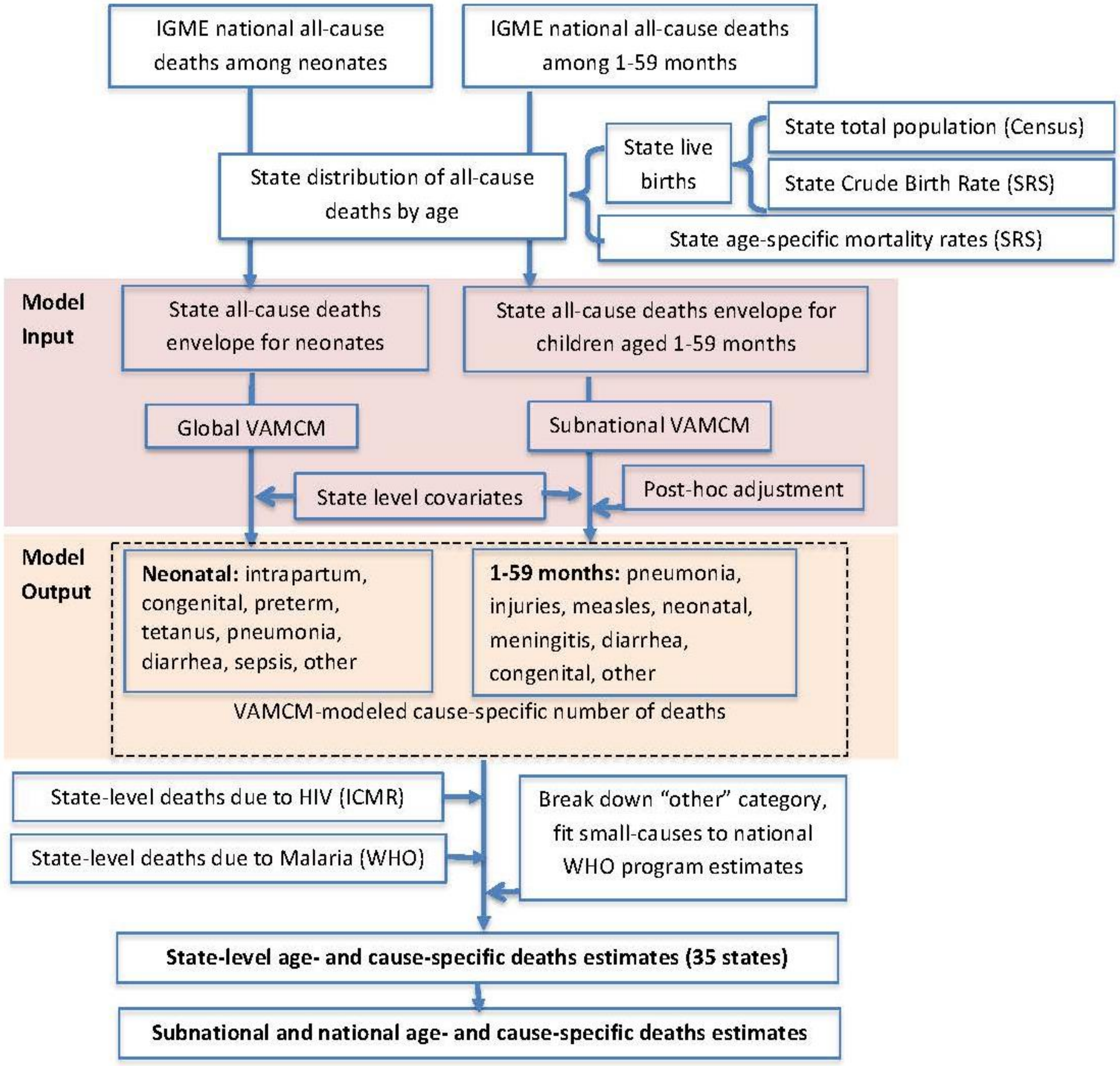

ICMR: Indian Council of Medical Research; IGME: Inter-agency Group for Child Mortality Estimation of United Nations; SRS: Sample Registration System; VAMCM: Verbal-Autopsy-based Multi-cause Model; WHO: World Health Organization.

Webappendix 4. State grouping

35 States	25 States	Region	EAGA	U5MR Strata
Andaman & Nicobar Islands	Other	South	No	Low
Andhra Pradesh & Telangana	Andhra Pradesh & Telangana	South	No	Low
Arunachal Pradesh	North East	Northeast	No	Medium
Assam	Assam	Northeast	Yes	Very high
Bihar	Bihar	East	Yes	Medium
Chandigarh	Other	North	No	Very low
Chhattisgarh	Chhattisgarh	Central	Yes	Medium
Dadra & Nagar Haveli	Other	West	No	Low
Daman & Diu	Other	West	No	Very low
Delhi	Delhi	North	No	Very low
Goa	Goa	West	No	Very low
Gujarat	Gujarat	West	No	Low
Haryana	Haryana	North	No	Low
Himachal Pradesh	Himachal Pradesh	North	No	Low
Jammu & Kashmir	Jammu & Kashmir	North	No	Low
Jharkhand	Jharkhand	East	Yes	Medium
Karnataka	Karnataka	South	No	Low
Kerala	Kerala	South	No	Very low
Lakshadweep	Other	South	No	Very low
Madhya Pradesh	Madhya Pradesh	Central	Yes	Very high
Maharashtra	Maharashtra	West	No	Very low
Manipur	North East	Northeast	No	Very low
Meghalaya	Meghalaya	Northeast	No	High
Mizoram	North East	Northeast	No	Medium
Nagaland	North East	Northeast	No	Very low
Odisha	Odisha	East	Yes	High
Puducherry	Other	South	No	Very low
Punjab	Punjab	North	No	Low
Rajasthan	Rajasthan	Central	Yes	Medium
Sikkim	North East	Northeast	No	Very low
Tamil Nadu	Tamil Nadu	South	No	Very low
Tripura	North East	Northeast	No	Low
Uttar Pradesh	Uttar Pradesh	Central	Yes	High
Uttarakhand	Uttarakhand	North	Yes	Low
West Bengal	West Bengal	East	No	Low

Webappendix 5. GATHER checklist

Item	Checklist item*	Section(s) or sources providing information
Objectives and funding		
1	Estimated indicator and population	Methods
2	Funding sources	Funding section of summary
Data Inputs		
For all data inputs from multiple sources that are synthesized as part of the study:		
3	Data identification	Methods and appendix 1-3; Methods and appendix of previous publication[13]
4	Inclusion/exclusion criteria	Methods and appendix of previous publications[13,29-32]
5	Included data sources and their main characteristics	Open access databases**
6	Potential important biases of input data	Discussion
For data inputs that contribute to the analysis but were not synthesized as part of the study:		
7	Source of other data inputs	Open access databases**
For all data inputs:		
8	Accessible input data files	Open access databases**
Data analysis		
9	Conceptual overview of the data analysis method	Methods and appendix of current and previous publications. [13,29-32] List of model covariates inspired by Mosley-Chen framework of child survival.
10	Description of all steps of the analysis	Methods and appendix 3. Methods of previous publications[13,29-32]
11	Model selection methods	Methods and appendix of current and previous publications [13,29-32]
12	Model performance and/or sensitivity analysis.	Methods and appendix 2
13	Uncertainty estimation methods	Methods; Methods and appendix of previous publications. [13,29-32]
14	Statistical code	Open access databases**
Results and discussion		
15	Accessible estimates data files	Open access databases**
16	Uncertainty of the estimates	Results and discussion
17	Results interpretation in light of existing evidence	Results and discussion
18	Limitations of the estimates	Discussion

*Detailed GATHER statement with explanation and elaboration of the items could be found on gather-statement.org

** Open access databases with input files and analytical code of the study could be accessed on the Maternal and Child Epidemiology Estimates project website: https://www.jhsph.edu/research/centers-and-institutes/institute-for-international-programs/current-projects/maternal-child-epidemiology-estimation/maternal-newborn-and-child-cause-of-death/index.html

Webappendix 6. Under-five cause of death distribution by region in India in 2015

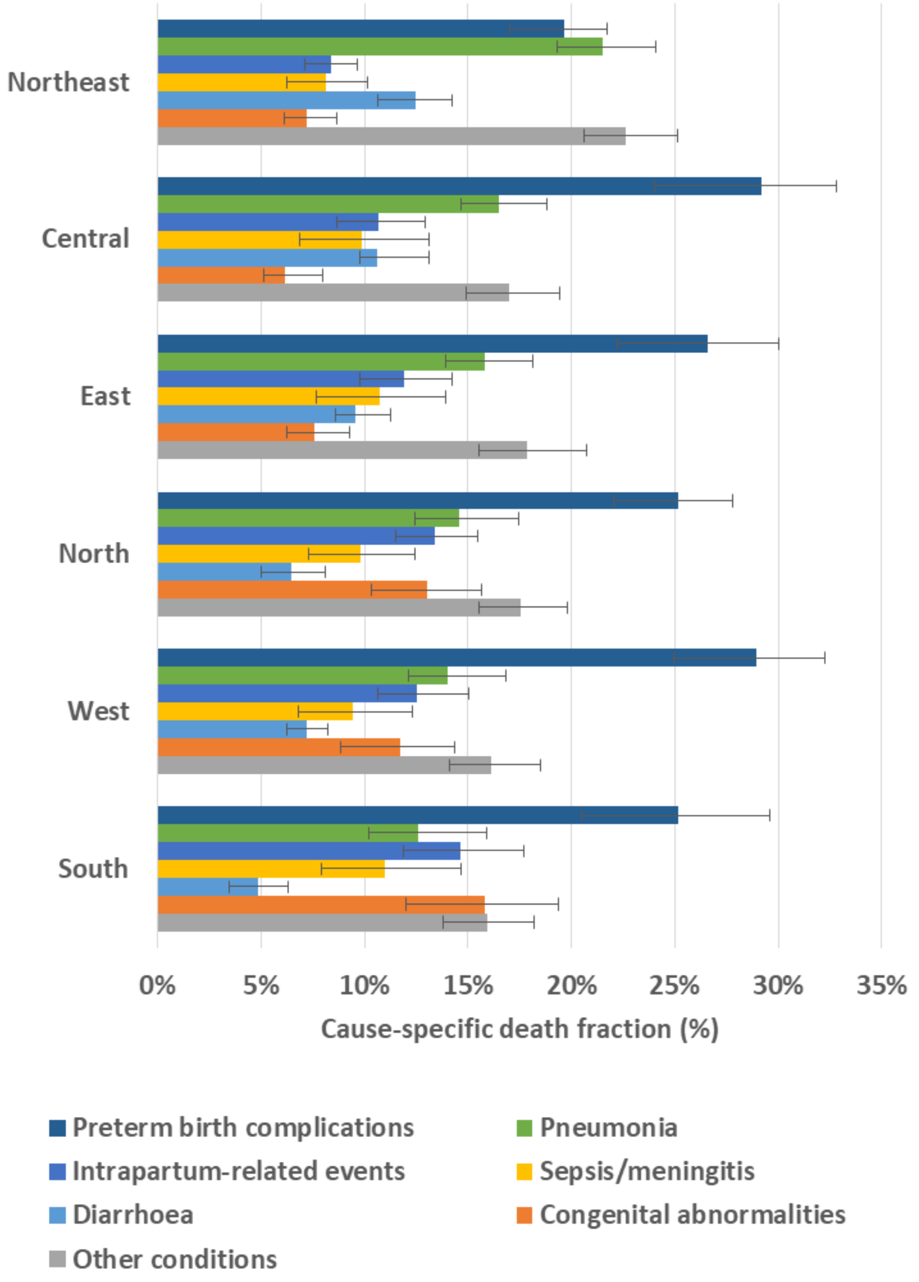

Note: Regions are ordered from top to bottom based on U5MR from high to low.

Webappendix 7. Under-five cause of death distribution in EAGA and non-EAG states in India in 2015

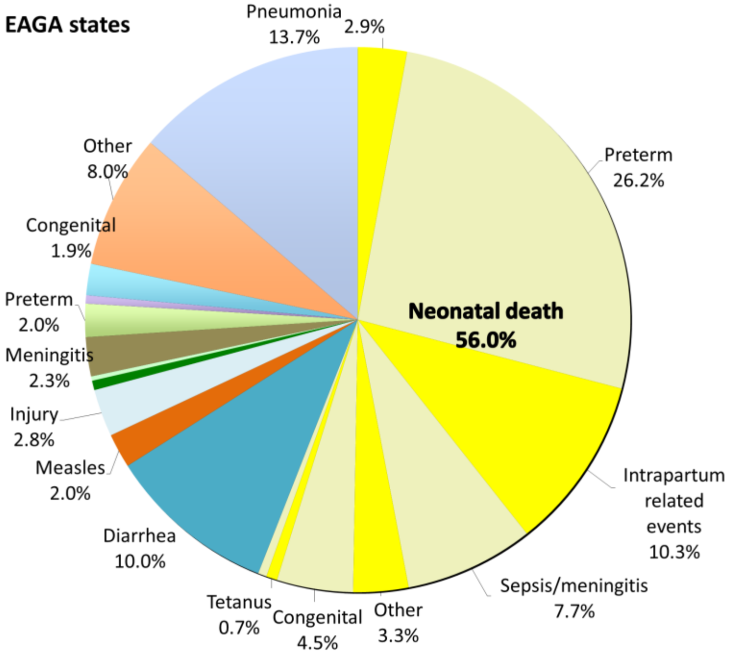

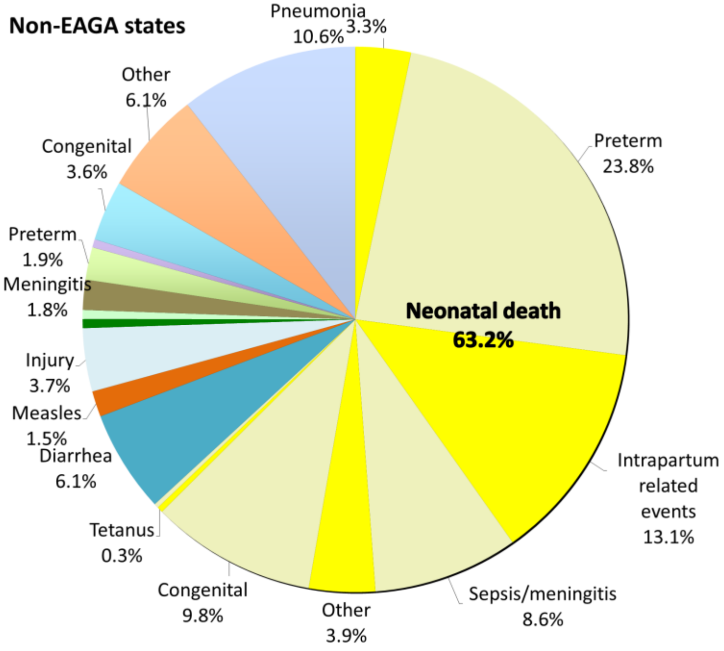

Webappendix 8. Under-five cause of death distribution in selected states in India in 2015

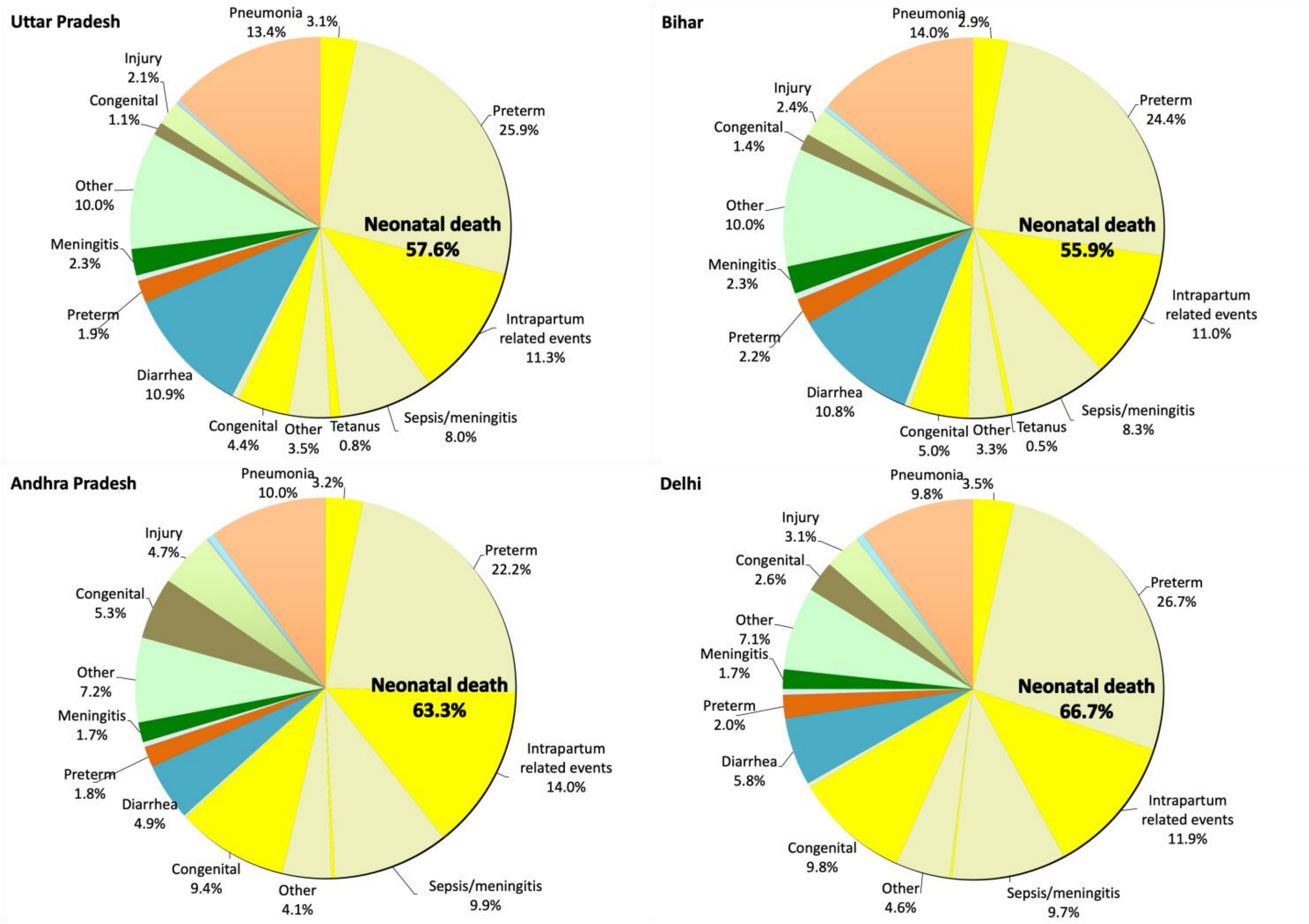

Webappendix 9. U5MR and NMR at the national and regional levels in India in 2000-2015

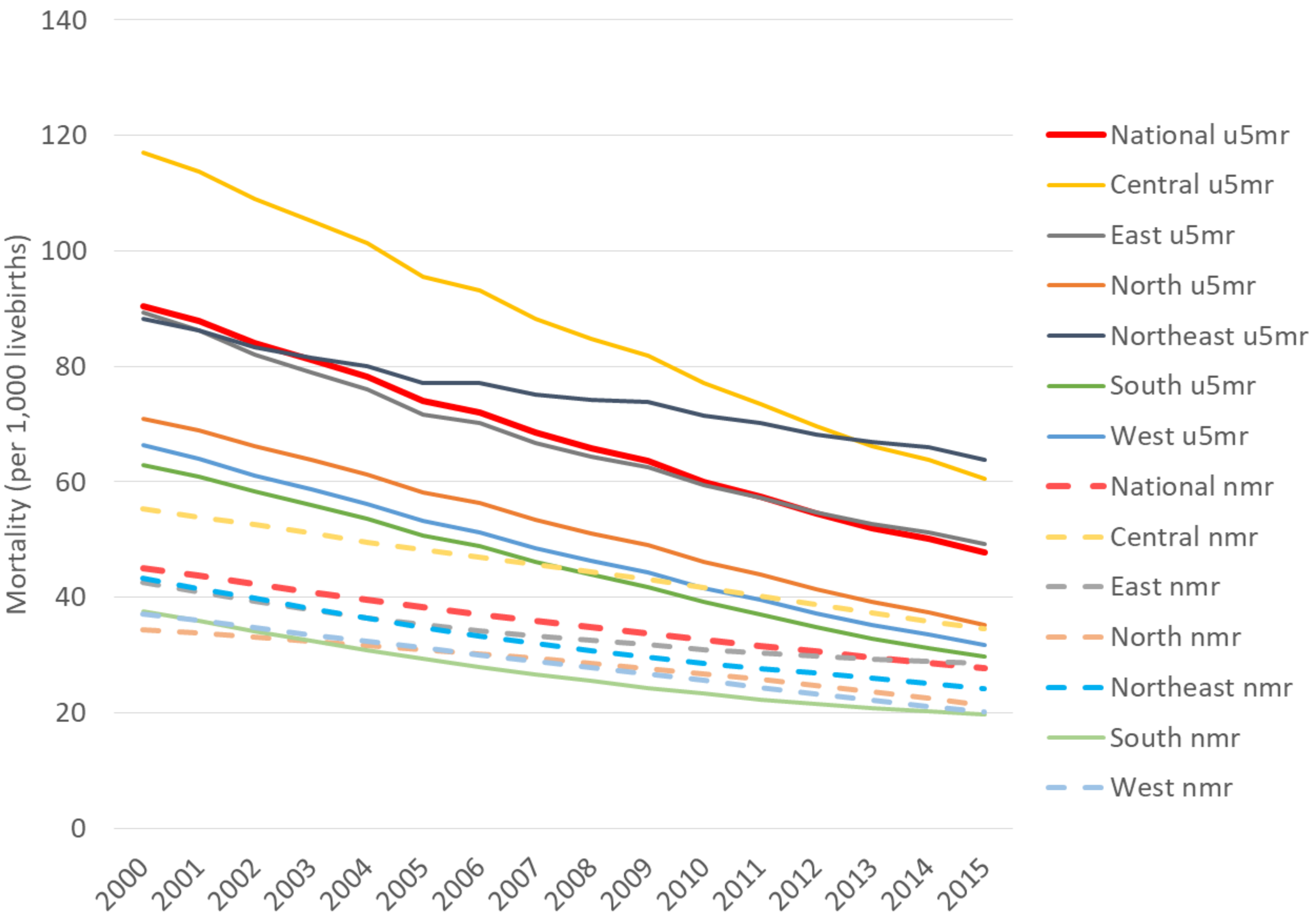

Webappendix 10. Ratio of U5MR between the Northeast and South Regions in India in 2000-2015

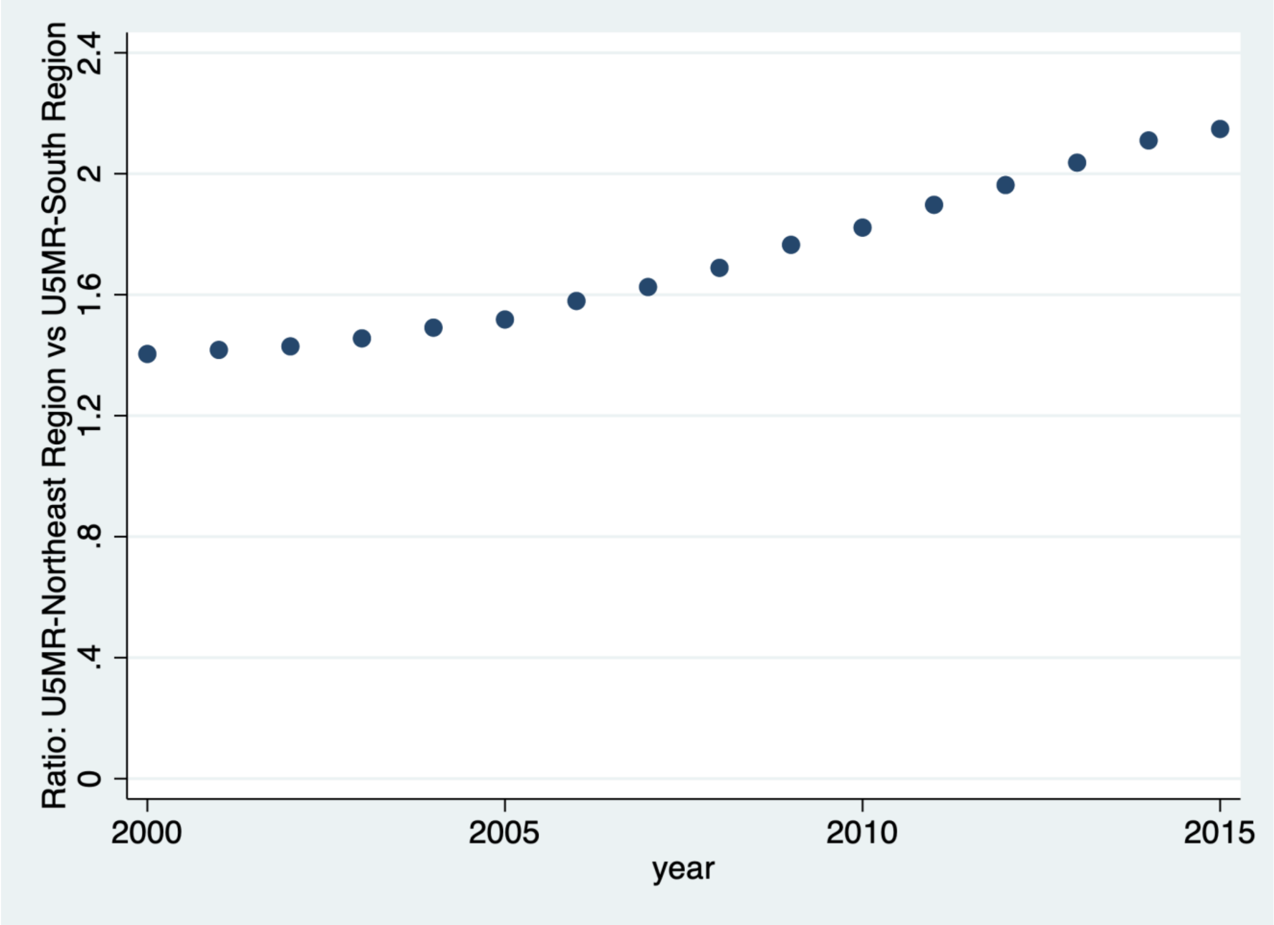

Webappendix 11. Annual rate of reduction (ARR) of U5MR, NMR, mortality rate among 1-59 month olds (PNMR) by state in India in 2000-2015

a) ARR of U5MR achieved in 2000-2015 and ARR needed to achieve SDG in 2015-2030 by state

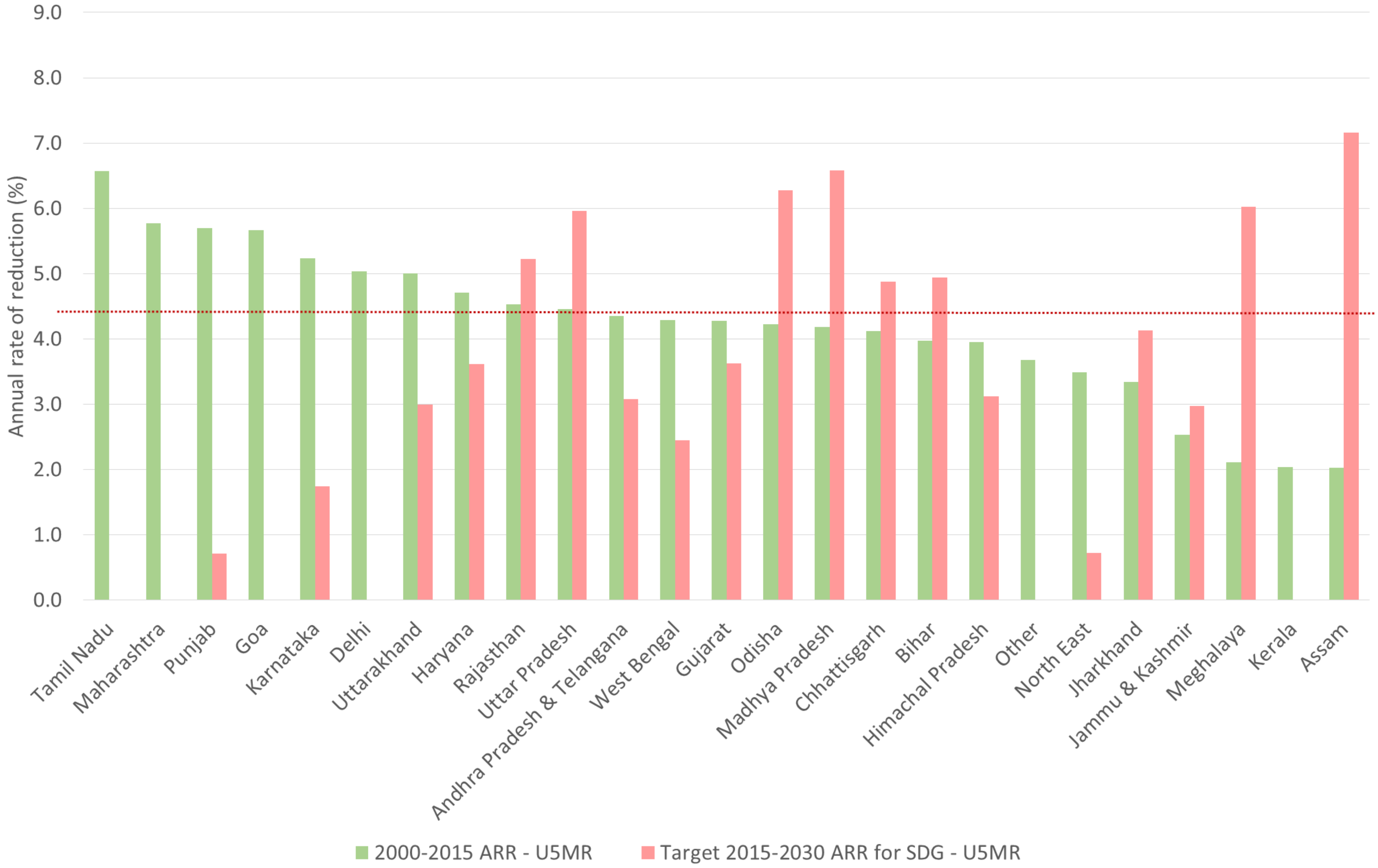

b) ARR of NMR in 2000-2015 and ARR needed to achieve the SDG NMR target in 2000-2030, by state

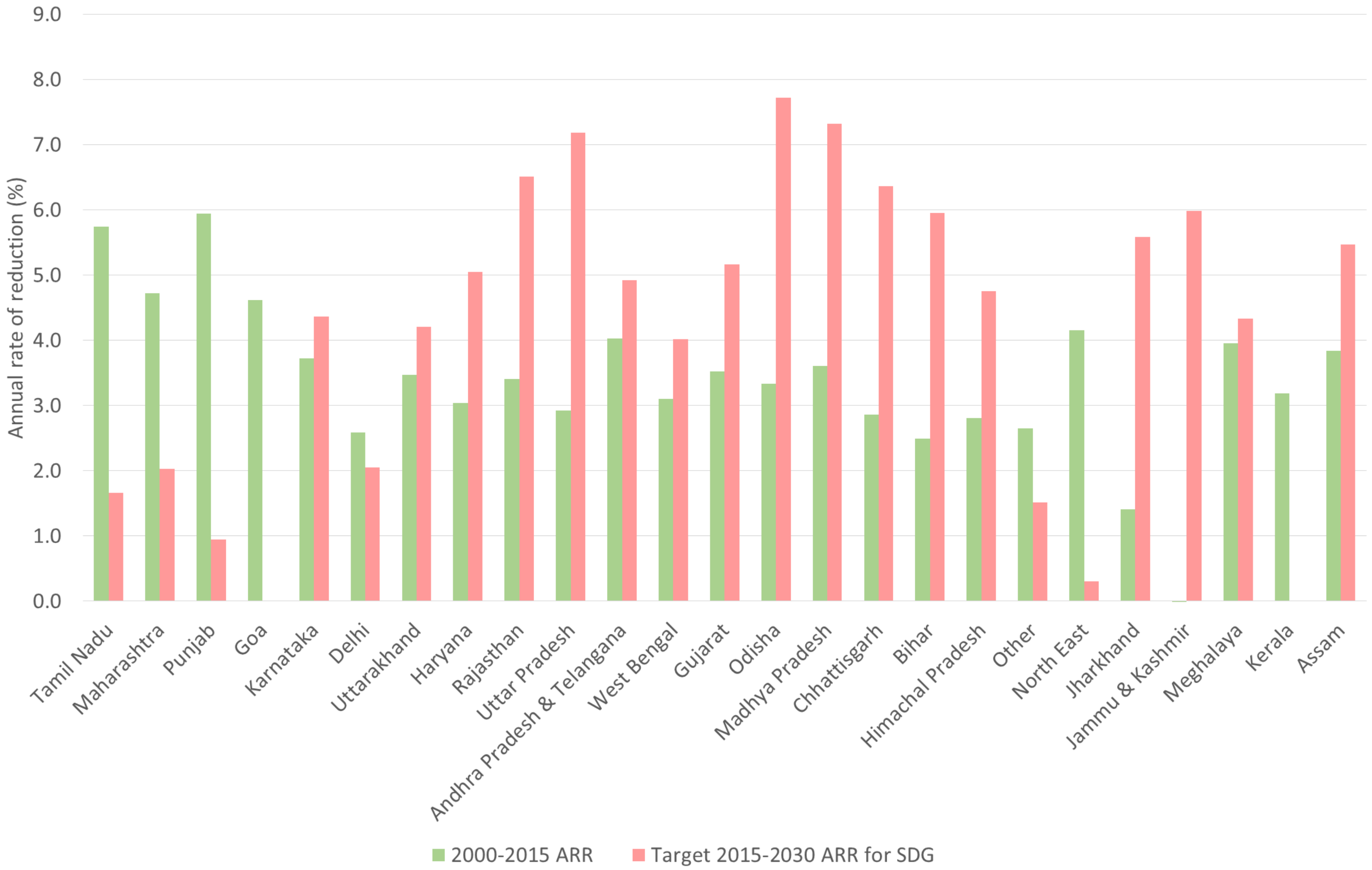

Note: Dotted red line represents ARR level required to achieve the MDG (4.4%). no red bar indicates that state has achieved the SDG U5MR target of at or below 25 per 1,000 livebirths or the SDG NMR target of at or below 12 per 1,000 livebirths in 2015.

Webappendix 12. National trends in cause-specific mortality fractions among a) neonates and b) children aged 1-59 months in India in 2000-2015

a) Neonates

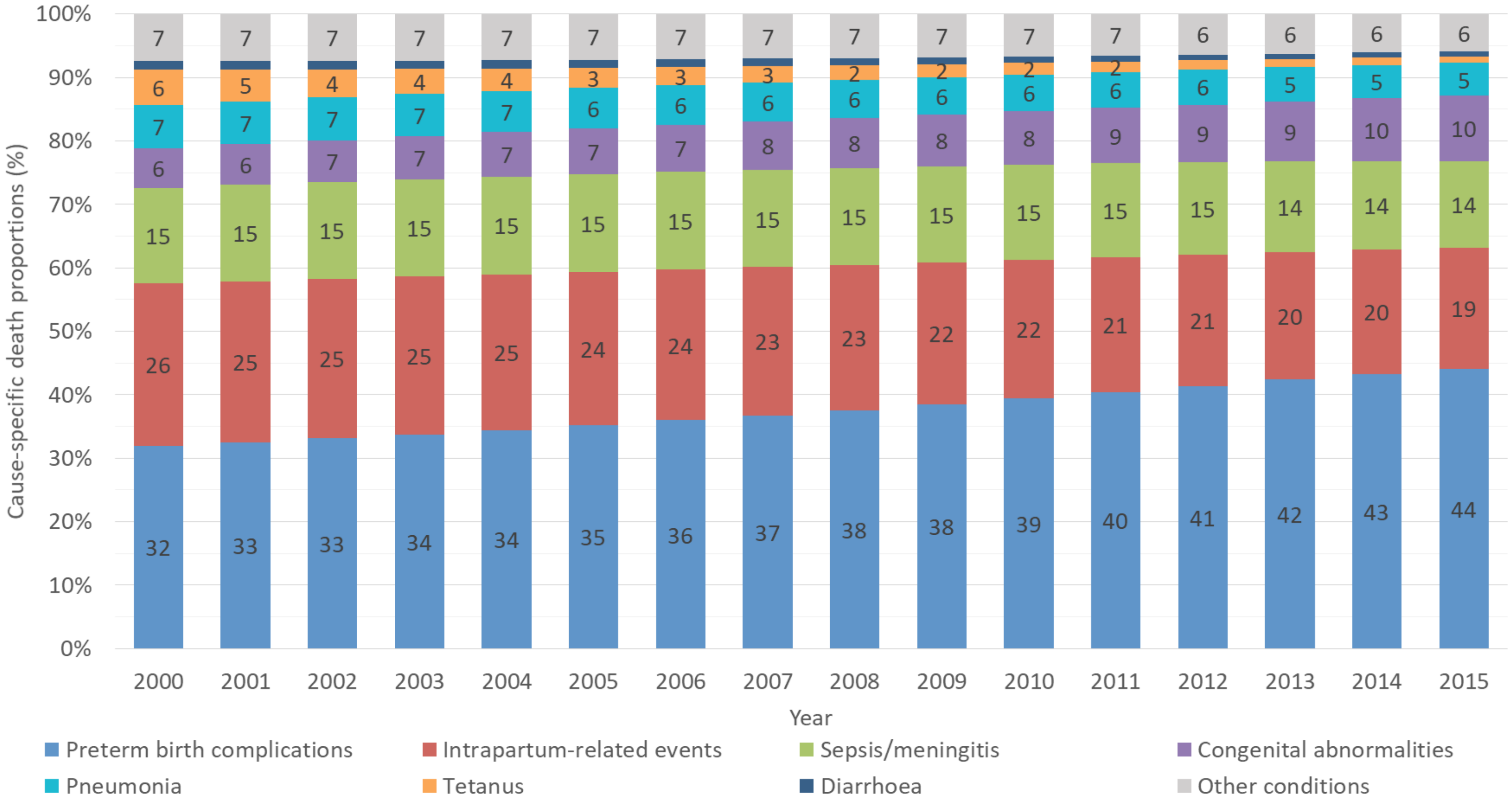

b) Children aged 1-59 months

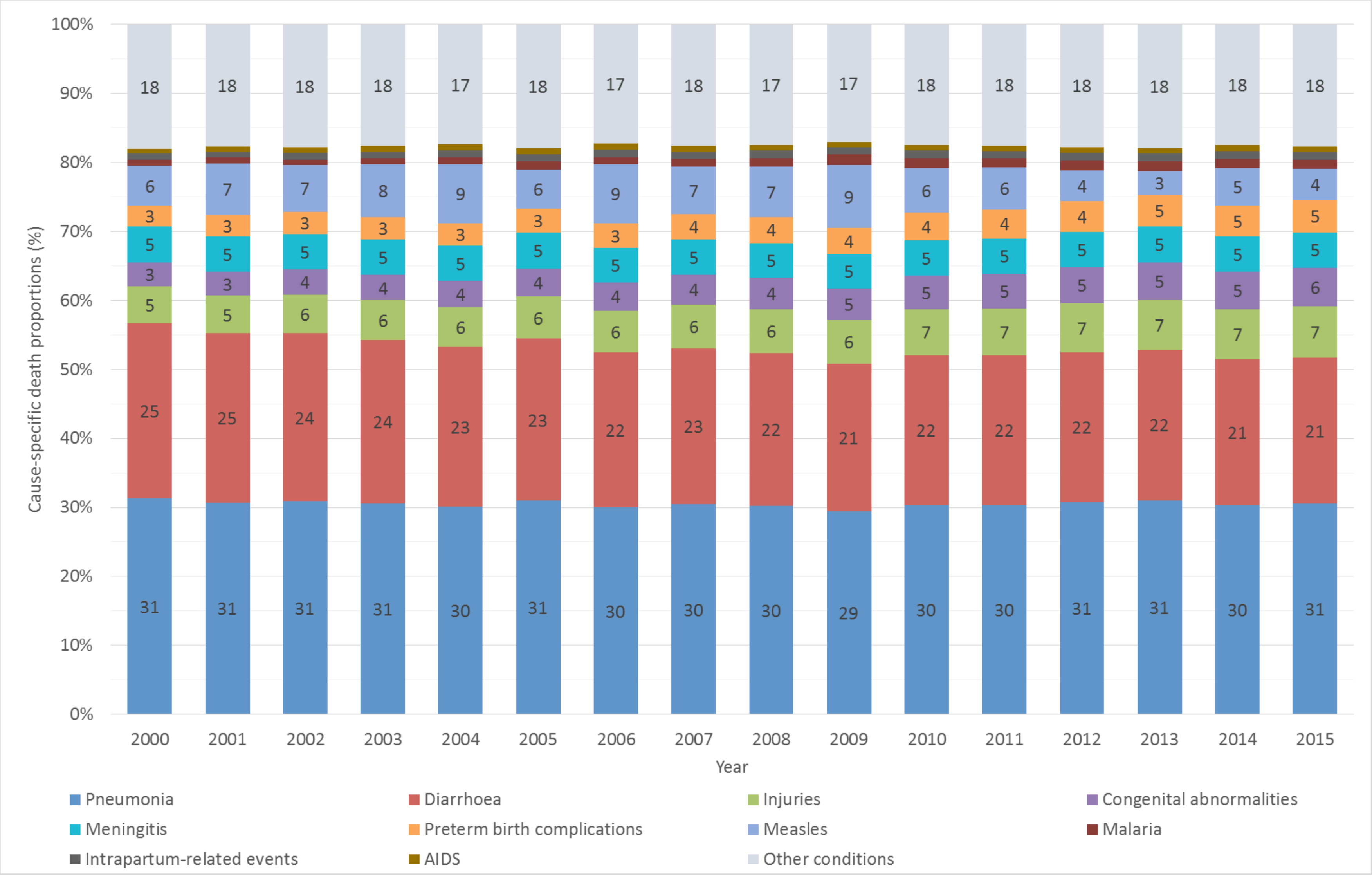

Webappendix 13. Annual rate of reduction in cause-specific mortality rates among neonates and children aged 1-59 months at national and regional level in India between 2000 and 2015

Cause of death	National	Northeast	Central	East	North	West	South
**Under-five**							
All causes	4.3	2.2	4.4	4.0	4.7	4.9	5.0
Diarrhoea	6.6	2.4	6.9	6.5	8.4	7.3	8.3
Pneumonia	5.5	1.6	5.7	5.5	6.6	6.3	6.4
Intrapartum-related events	5.1	5.6	5.4	4.8	4.2	5.6	5.0
Sepsis/meningitis	4.3	2.8	3.9	3.6	5.6	6.2	5.9
Preterm birth complications	1.2	1.1	0.6	0.4	2.4	2.9	4.0
Congenital abnormalities	0.6	-0.2	0.3	0.9	0.3	0.8	0.6
Other conditions	5.8	2.1	6.4	5.4	5.8	6.2	6.3
**0-27 days**							
All causes	3.2	3.9	3.2	2.7	3.2	4.1	4.3
Tetanus	14.8	16.4	15.8	13.9	9.2	11.3	14.0
Intrapartum-related events	5.2	5.9	5.5	4.9	4.2	5.6	5.0
Pneumonia	5.1	5.9	5.3	4.8	4.6	5.2	5.4
Sepsis/meningitis	3.9	3.9	3.2	3.0	5.1	6.1	5.7
Preterm birth complications	1.1	1.4	0.5	0.2	2.2	2.8	4.0
Congenital abnormalities	-0.1	2.6	-0.2	0.0	-1.3	0.1	-0.3
Other conditions	5.0	5.5	4.8	4.4	5.4	6.2	6.2
**1-59 months**							
All causes	5.4	0.9	5.8	5.4	6.5	6.2	6.2
Measles	7.1	3.1	7.4	7.8	7.0	6.8	7.2
Diarrhea	6.6	2.2	6.9	6.5	8.4	7.3	8.0
Meningitis	5.6	1.0	5.8	5.6	7.1	6.6	6.7
Pneumonia	5.6	0.9	5.8	5.6	7.1	6.6	6.7
Injuries	3.4	-1.7	3.2	3.9	4.5	4.5	4.1
Intrapartum-related events	3.4	0.1	2.8	3.6	5.4	5.1	5.1
Malaria	3.2	0.6	8.1	1.3	-16.4	-2.5	10.7
Preterm birth complications	2.5	-0.8	1.9	2.7	4.5	4.2	4.2
Congenital abnormalities	2.0	-3.3	1.5	3.0	2.8	2.7	2.6
Other conditions	5.5	0.9	5.7	5.3	6.9	6.8	7.1

Note: Negative ARR means increase in cause-specific mortality rate between 2000 and 2015.

Webappendix 14. Annual rate of reduction (ARR) of cause-specific mortality rates due to leading under-five causes by state in India between 2000 and 2015

a) Major infectious causes (diarrhea, pneumonia, sepsis/meningitis)

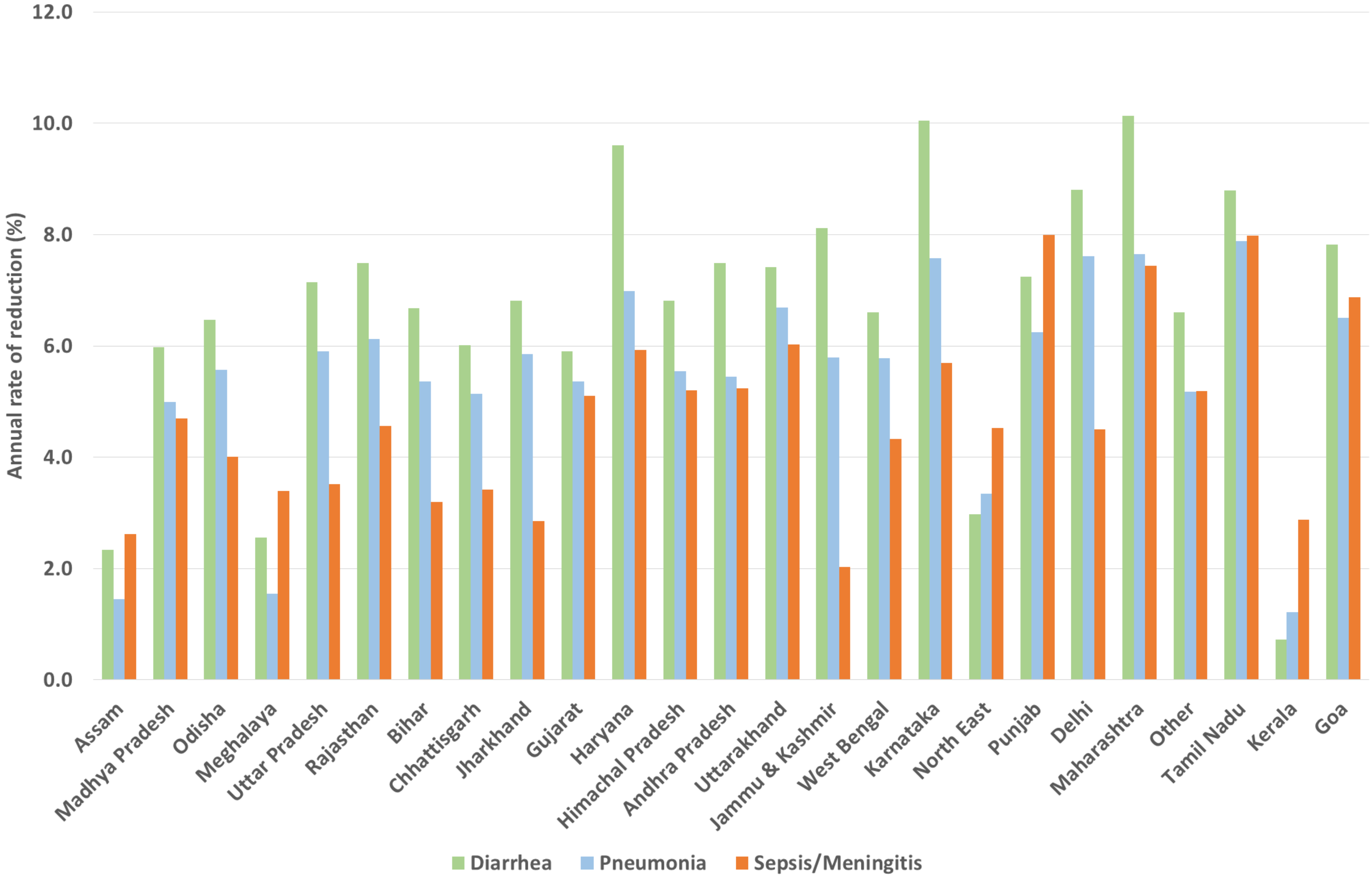

Note: States ordered by U5MR in 2015 with highest on the left and lowest on the right.

b) Major non-communicable diseases (preterm birth complications, intrapartum-related conditions and congenital abnormalities)

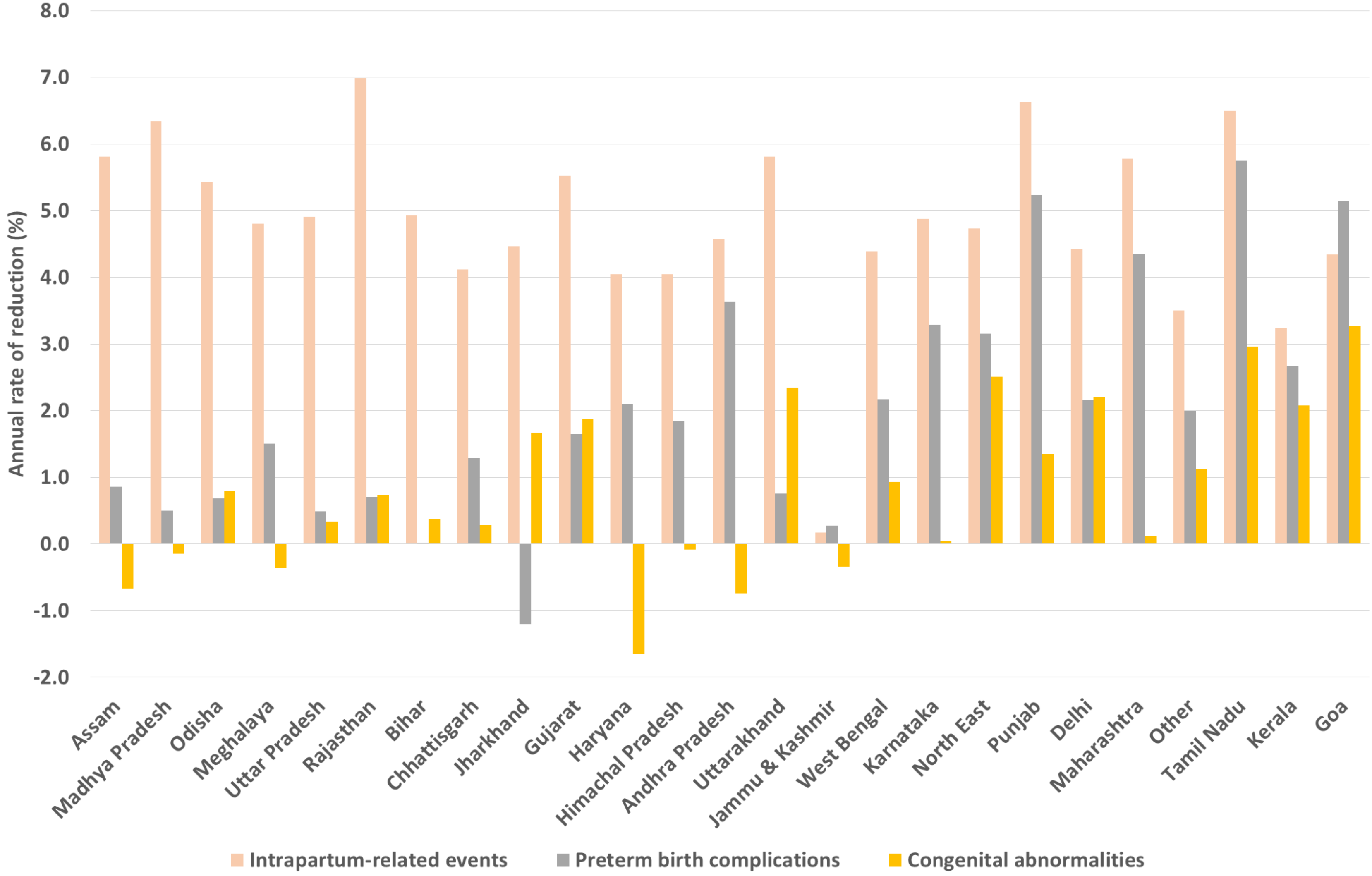

Note: States ordered based on U5MR in 2015 with highest on the left and lowest on the right.

Webappendix 15. Comparisons of all-cause mortality and live births estimates for year 2015 between the UN Inter-agency Group for Child Mortality Estimation (UN-IGME) and the Global Burden of Disease (GBD) Study

Estimates	Mortality rates (per 1,000 live births)			Under–5 deaths (thousand)	Implied live births (million)
	Neonatal	1-59 months	Under 5		
GBD [33]	23.1	19.2	41.7	942.7	22.6
UN-IGME [7]	26.4	20.2	46.6	1140.3	24.5

Webappendix 16. Comparison between MCEE, MDS and GBD of cause-specific mortality fractions of a) neonatal deaths and b) deaths among those aged 1-59 months in India in 2015

a) Cause-specific mortality fractions of neonatal deaths

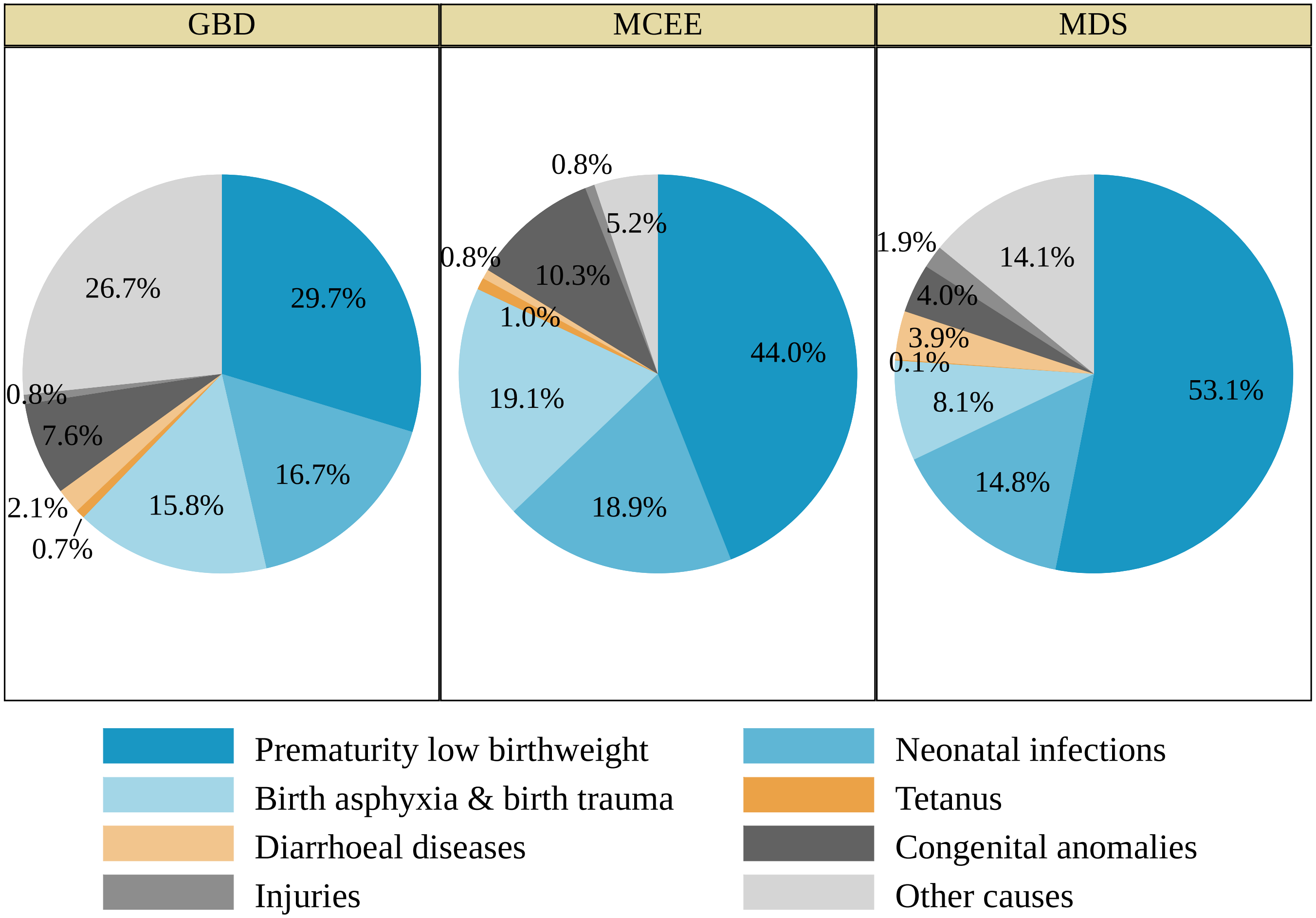

b) Cause-specific mortality fractions of deaths among those aged 1-59 months

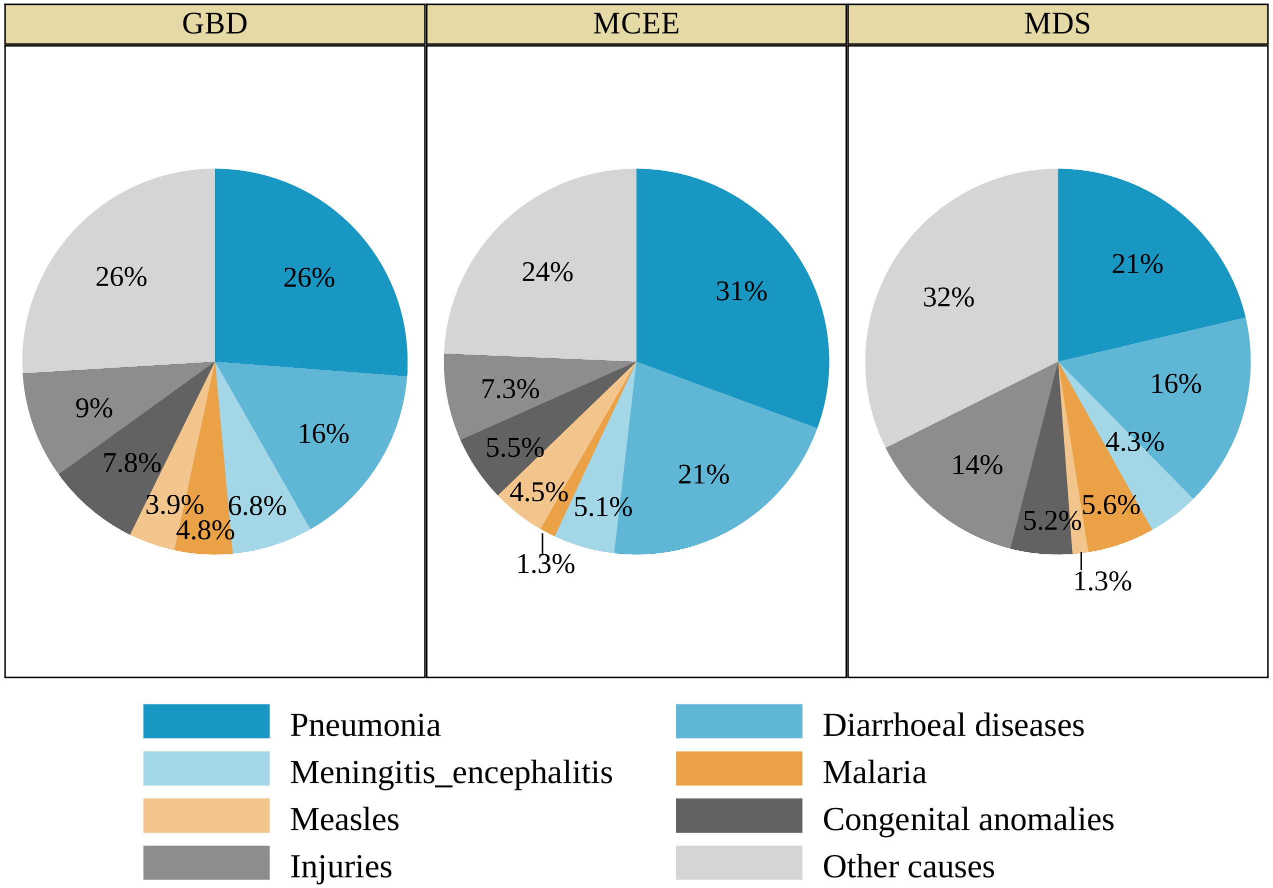

Webappendix 17. Comparisons between MCEE and MDS of state a) all-cause neonatal, b) 1-59-month deaths, and c) livebirths estimates in India in 2000-2015

a) All-cause neonatal deaths

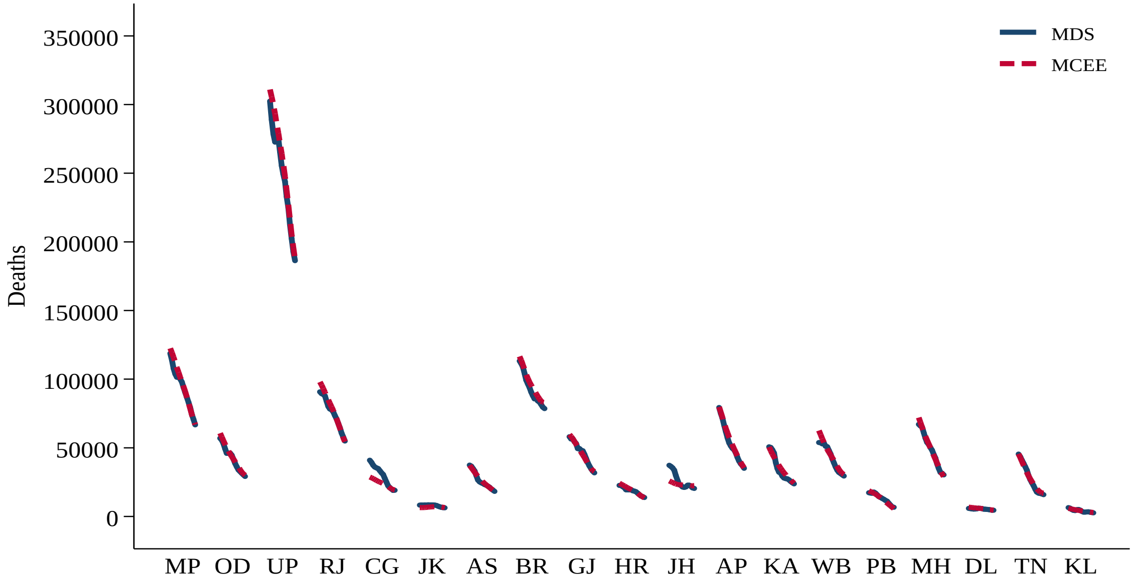

b) 1-59-month deaths

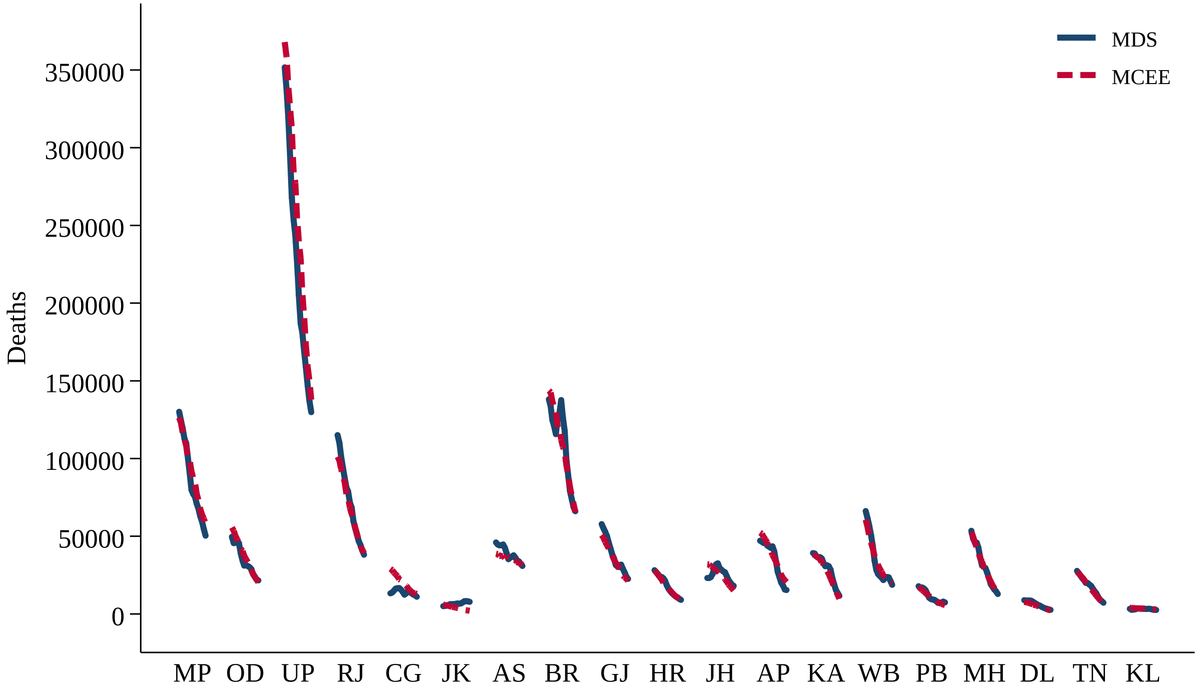

c) Livebirths

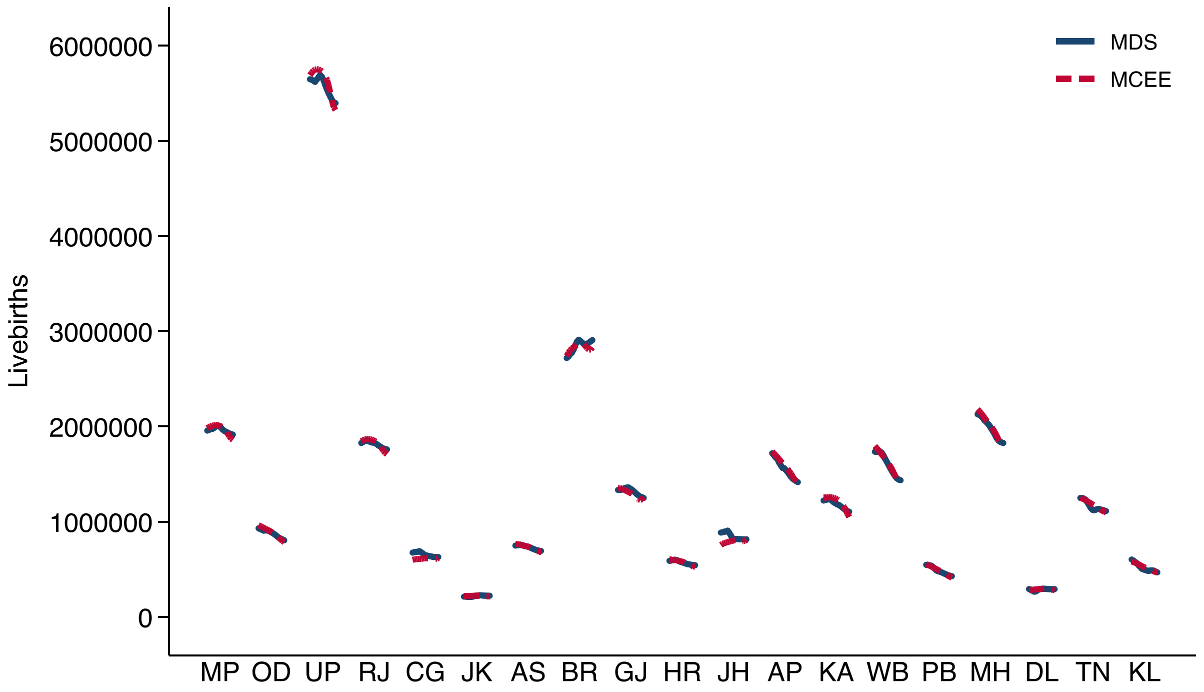

State abbreviations: Andaman and Nicobar (AN); Andhra Pradesh (AP); Arunachal Pradesh (AR); Assam (AS); Bihar (BR); Chandigar (CH); Chhattisgarh (CG); Dadra and Nagar Haveli (DN); Daman and Diu (DD); NCT of Delhi (DL); Goa (GO); Gujarat (GJ); Haryana (HR); Himachal Pradesh (HP); Jammu and Kashmir (JK); Jharkhand (JH); Karnataka (KA); Kerala (KL); Lakshadweep (LK); Madhya Pradesh (MP); Maharashtra (MH); Manipur (MN); Meghalaya (MG); Mizoram (MZ); Nagaland (NG); Odisha (OD); Puducherry (PD); Punjab (PB); Rajasthan (RJ); Sikkim (SK); Tamil Nadu (TN); Tripura (TP); Uttar Pradesh (UP); Uttarakhand (UK); West Bengal (WB).

Webappendix 18. Comparisons of trends in mortality rates due to a) the three leading causes of neonatal deaths and b) pneumonia and diarrhea in big states in India in 2000-2015

a) Three leading causes of neonatal deaths
Prematurity/low birthweight

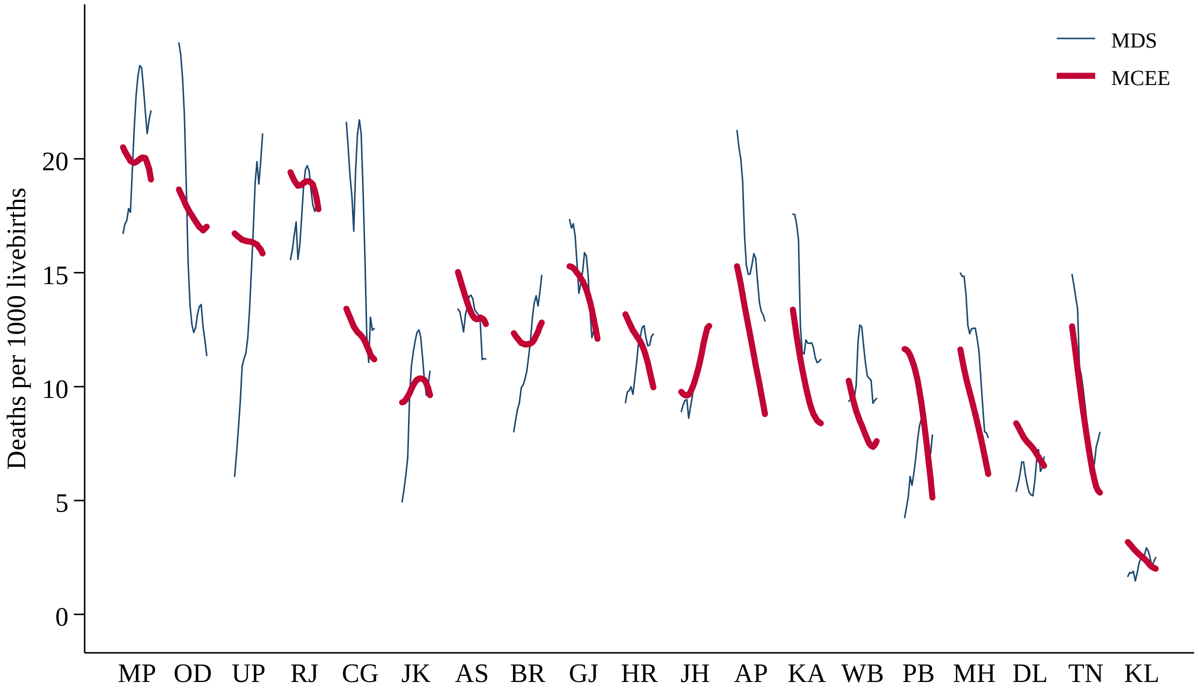

Neonatal infections

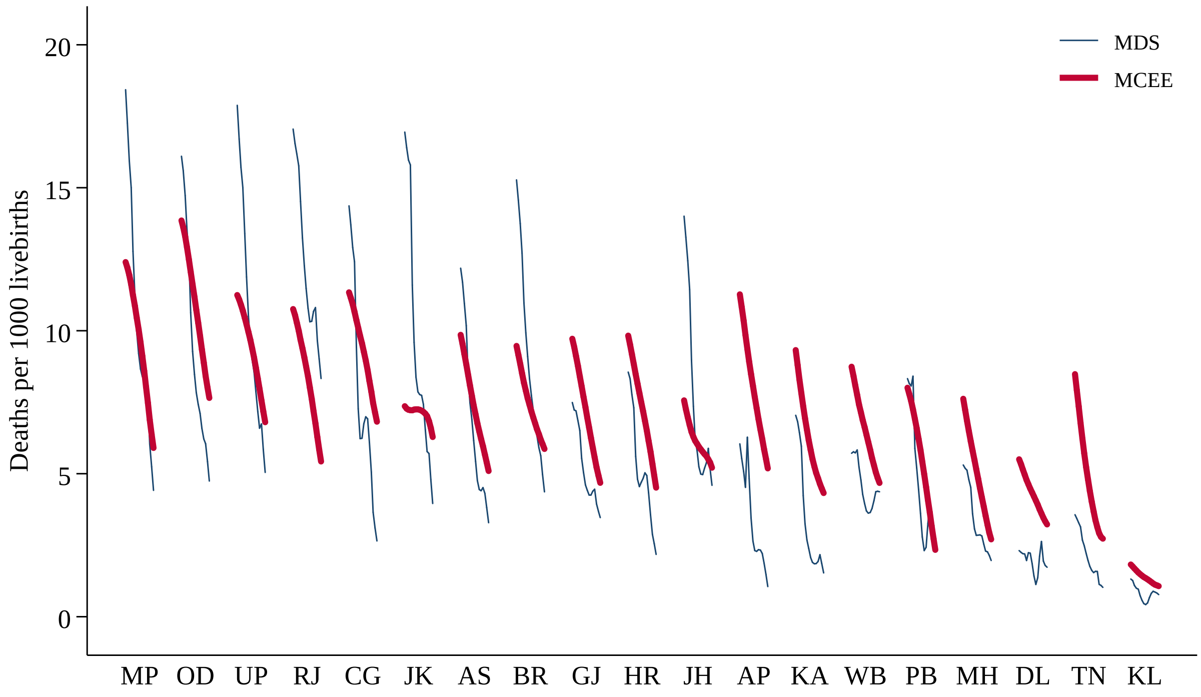

Birth asphyxia/trauma

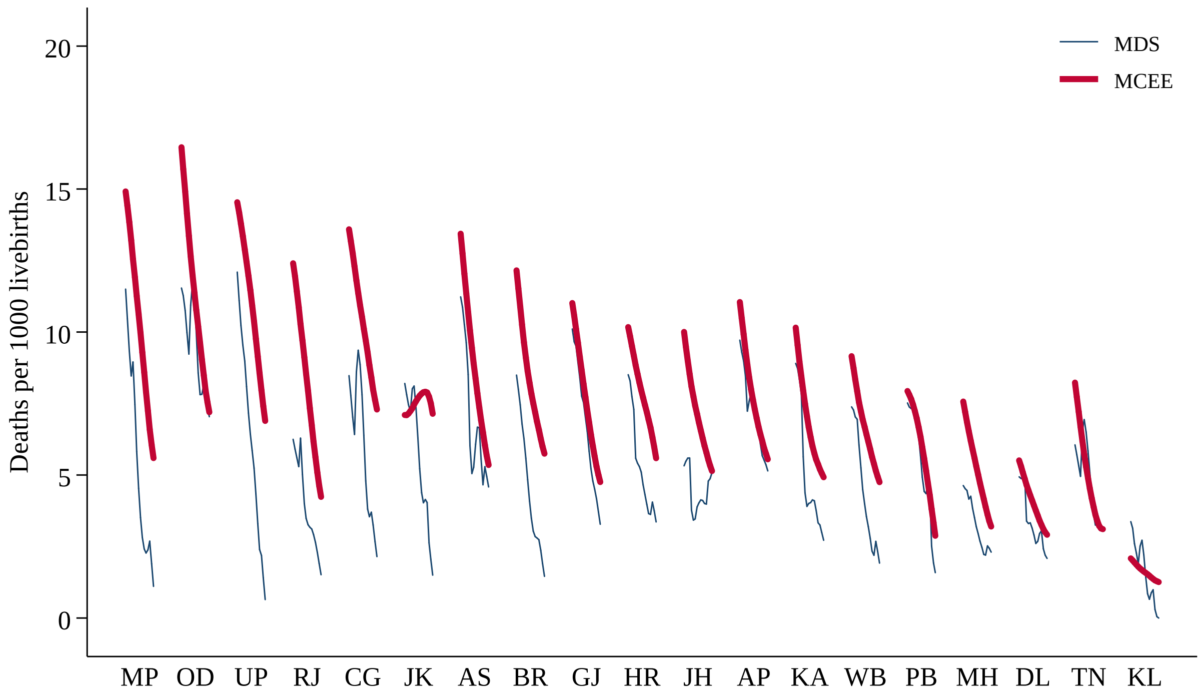

b) Pneumonia and diarrhea

Pneumonia

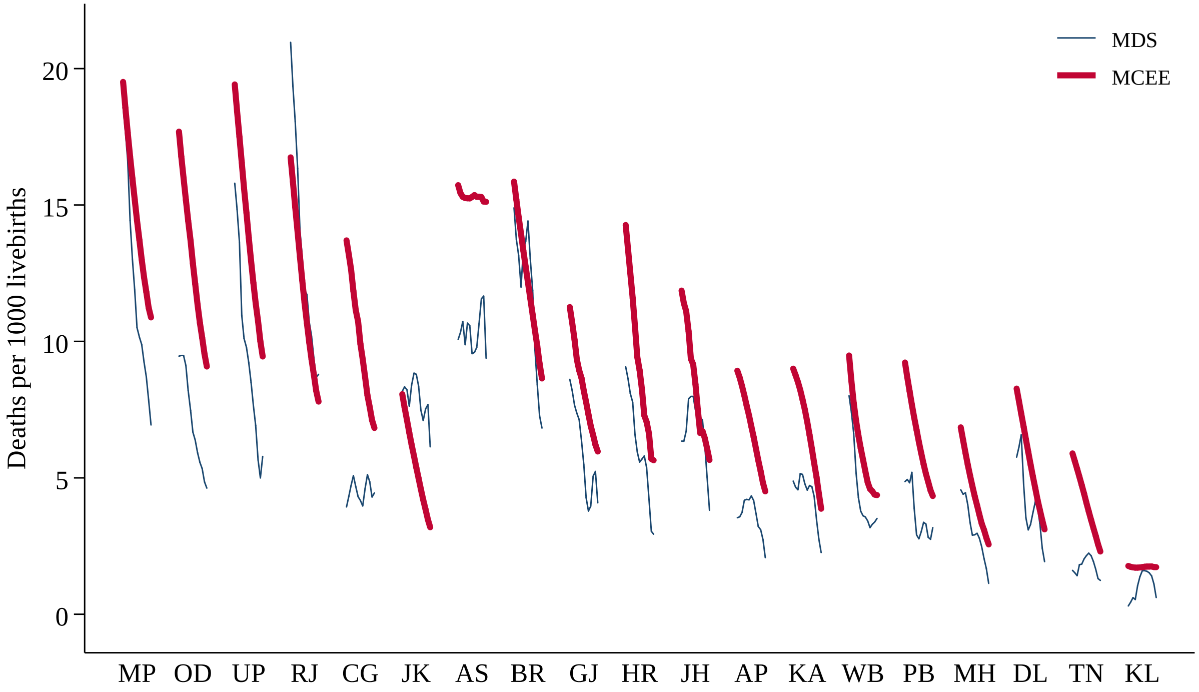

Diarrhea

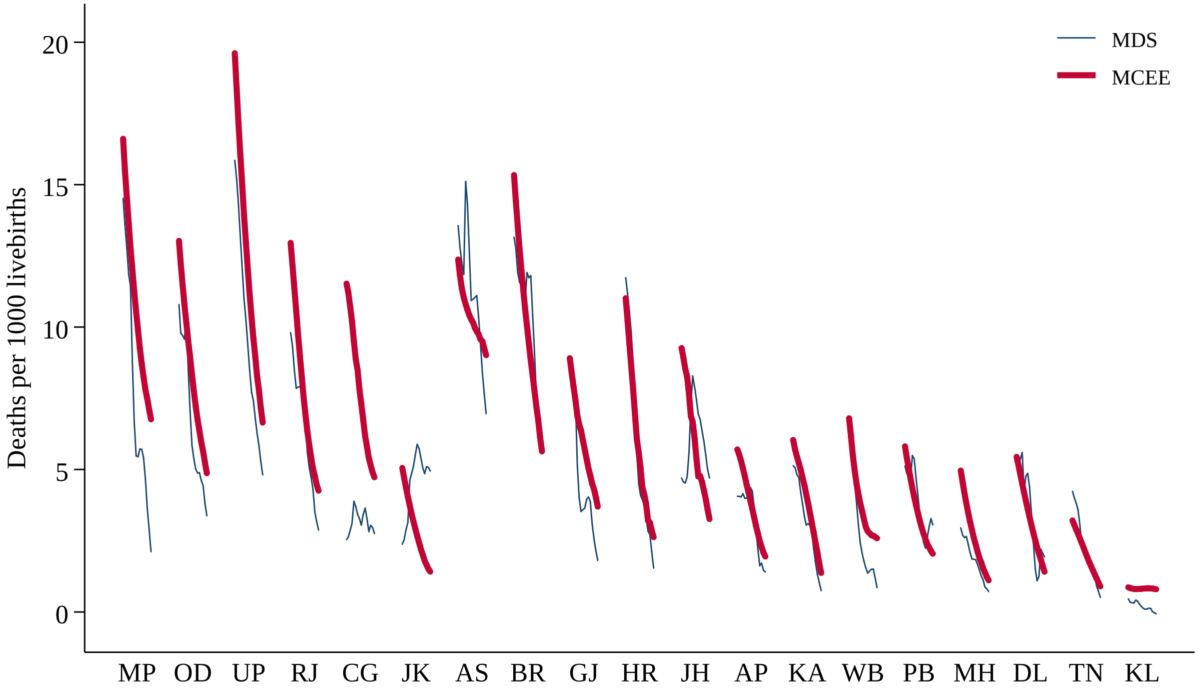

State abbreviations: Andaman and Nicobar (AN); Andhra Pradesh (AP); Arunachal Pradesh (AR); Assam (AS); Bihar (BR); Chandigarh (CH); Chhattisgarh (CG); Dadra and Nagar Haveli (DN); Daman and Diu (DD); NCT of Delhi (DL); Goa (GO); Gujarat (GJ); Haryana (HR); Himachal Pradesh (HP); Jammu and Kashmir (JK); Jharkhand (JH); Karnataka (KA); Kerala (KL); Lakshadweep (LK); Madhya Pradesh (MP); Maharashtra (MH); Manipur (MN); Meghalaya (MG); Mizoram (MZ); Nagaland (NG); Odisha (OD); Puducherry (PD); Punjab (PB); Rajasthan (RJ); Sikkim (SK); Tamil Nadu (TN); Tripura (TP); Uttar Pradesh (UP); Uttarakhand (UK); West Bengal (WB).

References

1. Registrar General of India. A-2 Decadal Variation In Population Since 1901.; 2016.
2. Registrar General of India. Compendium of India's Fertility and Mortality Indicators 1971-2007 based on the Sample Registration System. *Government of India, New Delhi* 2009.
3. Registrar General of India. Sample Registration System Statistical Reports (various years). New Delhi, India: Registrar General of India, 2014.
4. Registrar General of India. Sample Registration System Bulletin. New Delhi.: Vital Statistics Division, Registrar General of India, 2016.
5. Greville TN. Short methods of constructing abridged life tables. Mathematical Demography: Springer; 1977: 53-60.
6. United Nations Department of Economic and Social Affairs Population Division. World Population Prospects: The 2015 Revision, DVD Edition. 2015.
7. You D, Hug L, Ejdemyr S, et al. Global, regional, and national levels and trends in under-5 mortality between 1990 and 2015, with scenario-based projections to 2030: a systematic analysis by the UN Inter-agency Group for Child Mortality Estimation. *The Lancet* 2015; **386**(10010): 2275-86.
8. Stover J, Brown T, Marston M. Updates to the Spectrum/Estimation and Projection Package (EPP) model to estimate HIV trends for adults and children. *Sexually transmitted infections* 2012; **88**(Suppl 2): i11-i6.
9. Adam MA, Johnson LF. Estimation of adult antiretroviral treatment coverage in South Africa. *SAMJ: South African Medical Journal* 2009; **99**(9): 661-7.
10. Becquet R, Marston M, Dabis F, Zaba B, Newell M, Ghys P. Survival of children HIV-infected perinatally or through breastfeeding: a pooled analysis of individual data from sub-Saharan Africa. The 17th Conference on Retroviruses and Opportunistic Infections San Francisco, USA; 2010; 2010.
11. Marston M, Becquet R, Zaba B, et al. Net survival of perinatally and postnatally HIV-infected children: a pooled analysis of individual data from sub-Saharan Africa. *International journal of epidemiology* 2011: dyq255.
12. Marston M, Todd J, Glynn JR, et al. Estimating 'net'HIV-related mortality and the importance of background mortality rates. *Aids* 2007; **21**: S65-S71.
13. Liu L, Oza S, Hogan D, et al. Global, regional and national causes of child mortality in 2000-2015 – implications for the Sustainable Development Goals 2016.
14. Cibulskis RE, Aregawi M, Williams R, Otten M, Dye C. Worldwide incidence of malaria in 2009: estimates, time trends, and a critique of methods. *PLoS Med* 2011; **8**(12): e1001142.
15. World Health Organization. World malaria report 2008: World Health Organization; 2008.
16. Reserve Bank of India. Handbook of Statistics on Indian Economy. Mumbai, India: Reserve Bank of India; 2015.
17. Million Death Study Collaborators. Causes of neonatal and child mortality in India: a nationally representative mortality survey. *The Lancet* 2010; **376**(9755): 1853-60.
18. Davis S, Feikin D, Johnson HL. The effect of Haemophilus influenzae type B and pneumococcal conjugate vaccines on childhood meningitis mortality: a systematic review. *BMC Public Health* 2013; **13 Suppl 3**: S21.
19. Watt JP, Wolfson LJ, O'Brien KL, et al. Burden of disease caused by Haemophilus influenzae type b in children younger than 5 years: global estimates. *Lancet* 2009; **374**(9693): 903-11.
20. Wolfson L, O'Brien K, Watt J, Henkle E, Deloria-Knoll M, McCall N. Methods to estimate the global burden of disease due to Haemophilus influenzae type b and Streptococcus pneumoniae in children less than 5 years of age. *Lancet Web annex* 2009: 893-902.
21. Griffiths U, Clark A, Gessner B, et al. Dose-specific efficacy of Haemophilus influenzae type b conjugate vaccines: a systematic review and meta-analysis of controlled clinical trials. *Epidemiology and infection* 2012; **140**(08): 1343-55.

22. International Institute for Population Sciences. District Level Household Survey, 2007-08. In: International Institute for Population Sciences, editor. Mumbai, India; 2010.
23. International Institute for Population Sciences. District Level Household Survey, 2012-13. In: International Institute for Population Sciences, editor. Mumbai, India; 2014.
24. Office of the Registrar General and Census Commissioner. Annual health survey bulletin 2012-13. In: Ministry of Home Affairs GoI, editor. New Delhi, India; 2014.
25. Office of the Registrar General and Census Commissioner. Annual health survey bulletin 2011-12. In: Ministry of Home Affairs GoI, editor. New Delhi, India; 2013.
26. Office of the Registrar General and Census Commissioner. Annual health survey bulletin 2010-11. In: Ministry of Home Affairs GoI, editor. New Delhi, India; 2012.
27. International Institute for Population Sciences. India National Family Health Survey (NFHS-4), 2015-16. In: International Institute for Population Sciences, editor. Deonar, Mumbai, India: International Institute for Population Sciences,; 2017.
28. Preston S, Heuveline P, Guillot M. Demography: measuring and modeling population processes. 2000.
29. Liu L, Oza S, Hogan D, et al. Global, regional, and national causes of child mortality in 2000–13, with projections to inform post-2015 priorities: an updated systematic analysis. *The Lancet* 2015; **385**(9966): 430-40.
30. Liu L, Johnson HL, Cousens S, et al. Global, regional, and national causes of child mortality: an updated systematic analysis for 2010 with time trends since 2000. *The Lancet* 2012; **379**(9832): 2151-61.
31. Johnson HL, Liu L, Fischer-Walker C, Black RE. Estimating the distribution of causes of death among children age 1–59 months in high-mortality countries with incomplete death certification. *International journal of epidemiology* 2010; **39**(4): 1103-14.
32. Oza S, Lawn JE, Hogan DR, Mathers C, Cousens SN. Neonatal cause-of-death estimates for the early and late neonatal periods for 194 countries: 2000–2013. *Bulletin of the World Health Organization* 2014; **93**: 19-28.
33. GBD Causes of Death Collaborators. Global, regional, and national age-sex specific mortality for 264 causes of death, 1980–2016: a systematic analysis for the Global Burden of Disease Study 2016. *The Lancet* 2017.
